# Hypoxic transcription gene profiles under the modulation of nitric oxide in nuclear run on-microarray and proteomics

**DOI:** 10.1186/1471-2164-10-408

**Published:** 2009-09-02

**Authors:** Emeka I Igwe, Silke Essler, Natalie Al-Furoukh, Nathalie Dehne, Bernhard Brüne

**Affiliations:** 1 Institute of Biochemistry I/ZAFES, Faculty of Medicine, Goethe-University Frankfurt, Theodor-Stern-Kai 7, 60590 Frankfurt, Germany

## Abstract

**Background:**

Microarray analysis still is a powerful tool to identify new components of the transcriptosome. It helps to increase the knowledge of targets triggered by stress conditions such as hypoxia and nitric oxide. However, analysis of transcriptional regulatory events remain elusive due to the contribution of altered mRNA stability to gene expression patterns as well as changes in the half-life of mRNAs, which influence mRNA expression levels and their turn over rates. To circumvent these problems, we have focused on the analysis of newly transcribed (nascent) mRNAs by nuclear run on (NRO), followed by microarray analysis.

**Results:**

We identified 196 genes that were significantly regulated by hypoxia, 85 genes affected by nitric oxide and 292 genes induced by the cotreatment of macrophages with both NO and hypoxia. Fourteen genes (Bnip3, Ddit4, Vegfa, Trib3, Atf3, Cdkn1a, Scd1, D4Ertd765e, Sesn2, Son, Nnt, Lst1, Hps6 and Fxyd5) were common to all treatments but with different levels of expression in each group. We observed that 162 transcripts were regulated only when cells were co-treated with hypoxia and NO but not with either treatment alone, pointing to the importance of a crosstalk between hypoxia and NO. In addition, both array and proteomics data supported a consistent repression of hypoxia-regulated targets by NO.

**Conclusion:**

By eliminating the interference of steady state mRNA in gene expression profiling, we obtained a smaller number of significantly regulated transcripts in our study compared to published microarray data and identified previously unknown hypoxia-induced targets. Gene analysis profiling corroborated the interplay between NO- and hypoxia-induced signaling.

## Background

Hypoxia causes cellular stress. In order to survive cells turn on adaptive mechanisms to improve oxygen transport and to ensure sufficient cellular ATP supply [1]. Central to this adaptation is the transcription factor hypoxia-inducible factor-1 (HIF-1), which stimulates genes involved in angiogenesis, erythropoiesis and glycolysis [2-4]. HIF-1 consists of an O_2_-regulated α-subunit (HIF-1α) and a constitutively expressed β-subunit (HIF-1β). Under normoxic conditions HIF-1α is continuously degraded by a family of prolyl hydroxylase domain-containing enzymes (PHD1, PHD2, PHD3), which hydroxylate two proline residues (Pro-402 and Pro-564) in the oxygen-dependent degradation domain of HIF-1α [5,6]. This allows its recognition by the von Hippel-Lindau protein E3 ubiquitin ligase complex and proteasomal degradation [7,8]. Conversely, under hypoxia hydroxylation of HIF-1α is impaired, proteasomal degradation is offset thus, provoking its accumulation. HIF-1α is hydroxylated in an oxygen-dependent manner pointing to PHDs as the cellular oxygen sensors [9-11]. In addition to the regulation of HIF-1α protein stability, the transcriptional activity of HIF-1 is regulated by hydroxylation of Asn-803 in the C-terminal trans-activating domain of HIF-1α. An asparagyl hydroxylase known as factor inhibiting HIF (FIH) [1] catalyzes this modification and interferes with the transcriptional activity of HIF-1α by blocking cofactor binding, e.g. p300/CREB [12].

Besides hypoxia, nitric oxide (NO) and/or NO-derived species regulate HIF-1α abundance and activity. Under normoxic conditions, NO donors induce HIF-1α stabilization and transcriptional activation of HIF-1 target genes [13-15]. Mechanistically, NO-dependent inhibition of PHD activity accounts for HIF-1α protein stabilization [16], although increased synthesis mediated by phosphatidylinositol 3-kinase or mitogen-activated protein kinase has been noticed [14]. Paradoxically, under hypoxic conditions, NO appears to destabilize rather than to stabilize HIF-1α. Nitric oxide donors (DETA-NO, GSNO) decrease hypoxia-elicited HIF-1α stabilization and HIF-1 transcriptional activation [17-19]. It is suggested that mitochondria play a role in NO-mediated regulation of HIF-1α under hypoxia [20-23]. Hagen *et al*. proposed that inhibition of cytochrome c oxidase by NO during hypoxia reduced mitochondrial oxygen consumption, leaving more oxygen available for PHDs to regain activity thus, allowing HIF-1α degradation [21]. Furthermore, NO-derived species and/or reactive oxygen species have been suggested to destabilize HIF-1α by their ability to reactivate PHDs [24,25]. Recently, calcium induced activation of calpain were also implicated in the degradation of HIF-1α by NO under hypoxia, adding a layer of complexity to HIF-1 regulation [15]. Since hypoxia and NO modulate HIF-1α in different microenvironments, we were prompted to investigate whether the modulation of HIF-1α by hypoxia or NO would generate identical or restricted gene profiles. Using pairwise comparison of the gene profile data generated by cells treated with hypoxia and/or NO, we were able to study the influence of NO on the gene expression profile of hypoxia-induced genes, as well as that of hypoxia on NO-induced genes. Herein we have used a combined NRO and microarray approach to study the crosstalk between hypoxia/NO on the genetic profile of hypoxia- or NO-regulated genes in RAW 264.7 macrophages.

## Results and Discussion

### Transcripts generated by hypoxia and/or DETA-NO in macrophages

**Figure 1:**
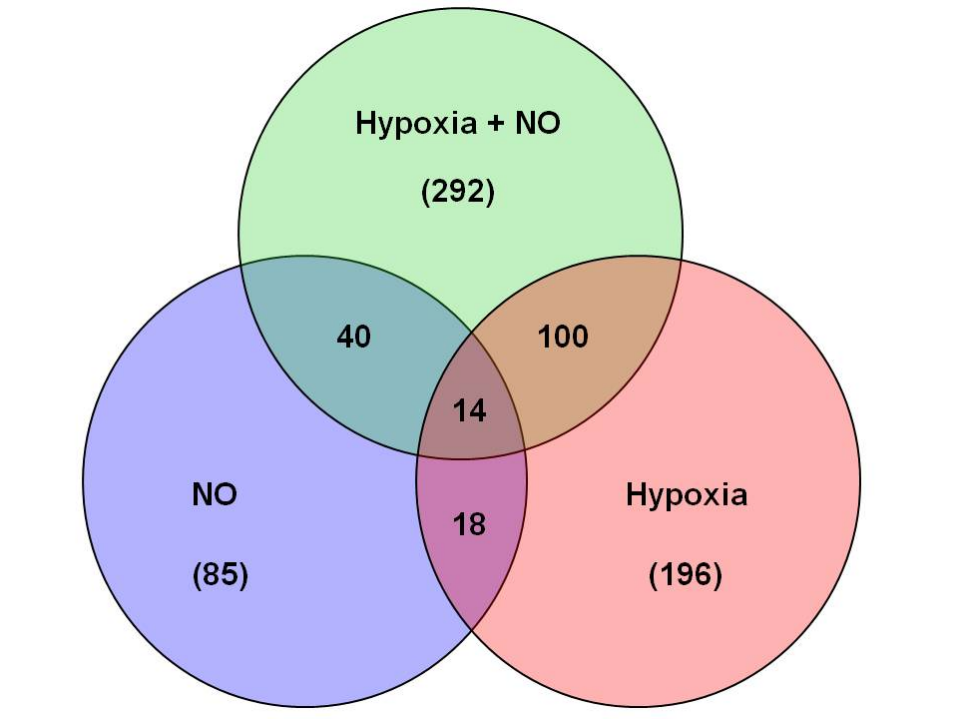
**Venn diagram of grouped genes**. Analysis of gene expression in RAW cells subjected to hypoxia (1% O_2_) and/or 0.5 mM DETA-NO for 6 h. After removing double and unknown transcripts data represent total number of genes regulated by each treatment (in parentheses) as well as overlapping genes shared by treatments. A complete list of genes for each treatment is provided in the supplementary tables 1-3. Detailed information of genes from the intersections is given in table 1-5.

The intention of this study was to identify hypoxia- or nitric oxide-regulated genes by a systematic analysis of *de novo *(nascent) transcription and to question whether regulation of HIF1α by hypoxia or nitric oxide would generate overlapping gene profiles. We chose to analyze newly synthesized mRNA obtained by NRO. This allowed detection of bona fide transcriptionally regulated genes, thus reducing the interference of steady-state mRNA turnover on the pool of expressed mRNA common to conventional microarray analysis. Therefore, RAW 264.7 cells were exposed to hypoxia (1% O_2_), DETA-NO (0.5 mM), or a combination of hypoxia and NO for 6 h. Transcriptional changes were systematically assessed using Affymetrix microarray analysis, which allowed following 32000 transcripts. [26]. Most gene transcripts (> 99% of genes on the array) remained unchanged by either hypoxia or NO, as well as in the combination of both. Only 196 genes were regulated by hypoxia with the majority being upregulated [see Additional file 1]. The greater number of the transcripts regulated by hypoxia is linked to energy consumption through glycolysis. Nitric oxide regulated a set of 85 genes shown in supplementary table 2 [see Additional file 2], while treatment of cells with both hypoxia and NO regulated 292 transcripts [see Additional file 3]. Figure 1 gives an overview of all groups with their number of regulated genes. Comparing gene profiles following treatments with hypoxia and/or NO, we observed that only 14 genes (Bnip3, Ddit4, Vegfa, Trib3, Atf3, Cdkn1a, Scd1, D4Ertd765e, Sesn2, Son, Nnt, Lst1, Hps6, and Fxyd5) were common to all treatments. It is not surprising that many of these genes have roles in cell death, DNA damage and apoptosis since under stress conditions cells try to avoid cell demise. Most of these genes had been previously described as hypoxia or NO regulated [27-29], underscoring the validity of this approach. Interestingly, 9 of the 14 common genes (Bnip3, Ddit4, Vegfa, Trib3, Atf3, Cdkn1a, Scd1, D4Ertd765e, Sesn2) were upregulated by hypoxia and/or NO, whereas 5 (Son, Nnt, Lst1, Hps6, and Fxyd5) were downregulated (Table 1). Most genes upregulated by hypoxia or NO were more strongly regulated under hypoxia, with the exception of Scd1 and Sesn2, whose levels were slightly higher with NO. For the commonly downregulated genes 4 out of the 5 identified ones were regulated to the same extent by hypoxia or NO. Interestingly, only one gene being downregulated (Fxyd5 a glycoprotein containing a Na^+^- K^+^-ATPase domain) revealed different levels of inhibition between hypoxia and NO, albeit being the strongest suppressed gene in the list. Hypoxia and NO downregulated Fxyd5 4.2-folds and 18.27-folds compared to controls. Contrary to the finding in this study, Fxyd5 has been shown to be upregulated in the mouse carotid body in response to 10% hypoxia [30], suggesting a different regulation pattern for this protein at different oxygen pressures. Fxyd5 affects the ion transport system of the blood brain barrier and modulates Na^+ ^absorption during cystic fibrosis [31]. The notion that NO decreased chloride adsorption by reducing Na^+^-K^+^-2Cl^- ^cotransporter activity and inhibits transepithelial ion movement in cystic fibrosis [32] corroborates our finding on repression of Fxyd5. Fxyd5 interacts with the Na^+^-K^+^-ATPase and modulates its properties after NO-treatment [33].

**Table 1:** 14 Genes common to all treatments; hypoxia, NO and hypoxia plus NO for 6 h.

**Symbol**	**Gene name**	**Ac**	**NO**	**Hyp**	**Hyp + NO**
Bnip3	BCL2/adenovirus E1B 19 kDa-interacting protein 1, NIP3	NM_009760.2	2.53	33.40	20.06

Ddit4	DNA-damage-inducible transcript 4	NM_029083.1	5.19	19.68	35.21

Vegfa	vascular endothelial growth factor A	NM_009505.2	2.06	9.08	6.55

Trib3	tribbles homolog 3 (Drosophila)	NM_144554.1	5.85	8.05	27.11

Atf3	activating transcription factor 3	NM_007498.2	2.37	7.27	7.26

Cdkn1a	cyclin-dependent kinase inhibitor 1A (P21)	NM_007669.2	2.07	2.89	4.25

Scd1	stearoyl-Coenzyme A desaturase 1	NM_009127.2	4.18	2.54	3.55

D4Ertd765e	DNA segment, Chr 4, ERATO Doi 765, expressed	NM_026728.1	2.91	2.52	2.30

Sesn2	sestrin 2	NM_144907.1	3.47	2.32	5.23

Son	Son cell proliferation protein	NM_178880.3	-2.06	-2.16	-2.23

Nnt	nicotinamide nucleotide transhydrogenase	AK087064.1	-2.04	-2.39	-2.41

Lst1	leukocyte specific transcript 1	NM_010734.1	-2.26	-2.62	-2.55

Hps6	Hermansky-Pudlak syndrome 6	NM_176785.1	-4.86	-3.74	-4.21

Fxyd5	FXYD domain-containing ion transport regulator 5	BC031112.1	-18.27	-4.20	-9.71

This list was obtained by comparing profiles of genes detected by the three treatments, excluding duplicate transcripts, unknown transcripts and transcripts not detected in one or two of the treatments. NO = nitric oxide (0.5 mM DETA-NO); Hyp = hypoxic treatment (1% O_2_).

In table 2 we listed a total of 18 genes common to macrophages treated with hypoxia or NO alone. The 18 transcripts consist of the 14 transcripts found in all treatments (Table 1) plus additional 4 transcripts (Frat2, CD52, Mafb and Ccl3). Eleven of the 18 genes were upregulated and 7 were downregulated by NO, while 10 genes were upregulated and 8 were downregulated by hypoxia. Interestingly in this category we identified Ccl3 (C-C motif) ligand 3, known as macrophage inflammatory protein 1 alpha (MIP1α), to be downregulated by hypoxia but upregulated by NO. Recent studies have shown that hypoxia preferentially regulates the accumulation of mononuclear phagocytes in hypoxic/ischemic areas [34,35]. Our data show that NO alone upregulated Ccl3 twofold compared to untreated cells, whereas hypoxia downregulated Ccl3 twofold. This finding correlates well with the report that hypoxia downregulates several members of the C-C ligand chemokine family including Ccl3 (MIP1α), MIP1β or Ccl2 (MCP-1) [36] or Ccl15, Ccl18 Ccl19 and Ccl23 [34]. Considering that chemokine signaling regulates monocyte/macrophage recruitment to inflammatory sites downregulation of these mediators by hypoxia may be necessary to prevent cell death and to limit the influx of pro-inflammatory cytokine producing cells. In contrast, NO with its pro- and anti-inflammatory properties upregulated Ccl3. Nitric oxide was shown to be coregulated with the chemokine MCP-1 in Kupffer cells treated with LPS [37] but a causative role for NO in MCP-1 regulation could not be established. However, there is evidence that NO is required for LPS-induced MCP-1 or MRP1 expression [38]. It is important that Ccl3, regulated either by hypoxia or NO alone, showed no expression regulation when cells were subjected to both treatments. This observation indicates a complex interplay between hypoxia and NO, with signal elimination under hypoxia/NO.

**Table 2:** 18 Genes common to treatments with hypoxia or nitric oxide.

**Symbol**	**Gene Name**	**Ac**	**NO**	**Hyp**
Bnip3	BCL2/adenovirus E1B 19 kDa-interacting protein 1, NIP3	NM_009760.2	2.53	33.40

Ddit4	DNA-damage-inducible transcript 4	NM_029083.1	5.19	19.68

Vegfa	vascular endothelial growth factor A	NM_009505.2	2.06	9.08

Trib3	tribbles homolog 3 (Drosophila)	NM_144554.1	5.85	8.05

Atf3	activating transcription factor 3	NM_007498.2	2.37	7.27

Cdkn1a	cyclin-dependent kinase inhibitor 1A (P21)	NM_007669.2	2.07	2.89

Scd1	stearoyl-Coenzyme A desaturase 1	NM_009127.2	4.18	2.54

D4Ertd765e	DNA segment, Chr 4, ERATO Doi 765, expressed	NM_026728.1	2.91	2.52

Frat2	frequently rearranged in advanced T-cell lymphomas 2	NM_177603.1	2.15	2.49

Sesn2	sestrin 2	NM_144907.1	3.47	2.32

Cd52	CD52 antigen	NM_013706.1	-2.02	-2.15

Son	Son cell proliferation protein	NM_178880.3	-2.06	-2.16

Mafb	v-maf musculoaponeurotic fibrosarcoma oncogene family, protein B (avian)	NM_010658.2	-2.49	-2.32

Nnt	nicotinamide nucleotide transhydrogenase	AK087064.1	-2.04	-2.39

Ccl3	chemokine (C-C motif) ligand 3	X12531.1	2.22	-2.42

Lst1	leukocyte specific transcript 1	NM_010734.1	-2.26	-2.62

Hps6	Hermansky-Pudlak syndrome 6	NM_176785.1	-4.86	-3.74

Fxyd5	FXYD domain-containing ion transport regulator 5	BC031112.1	-18.27	-4.20

Gene transcripts regulated by hypoxia (1% O_2_) or NO (0.5 mM DETA-NO) were analyzed to identify overlapping genes in these set of data. Hyp = hypoxia treatment; NO = nitric oxide treatment.

Considering that HIF-1 regulates a number of genes under hypoxia [39] or in response to NO, we were interested to examine opposing regulation patterns. Therefore, we analyzed to what extent hypoxia affects the gene profile generated by NO. We compared profiles of cells treated with NO to cells treated with NO plus hypoxia (Table 3). We identified 40 genes in response to NO or hypoxia plus NO. The patterns of gene profiles were similar for both treatments, though significant differences exist in the expression levels of some transcripts in response to NO or hypoxia/NO. Thirty of the transcripts (75%) were upregulated and only 10 (25%) were downregulated by both treatments. However, we noticed gene specific regulation when both data were compared. The majority of the upregulated genes (17 genes; 56.7%) revealed no changes in their expression when hypoxia was added to NO while 12 transcripts (40%) were increased 2- to 6-fold, compared to treatments with NO alone. This suggests an additive effect cause by hypoxia on the expression of these transcripts. Amongst the commonly upregulated genes only one transcript (Scd1) was moderately upregulated when cells were cotreated with hypoxia plus NO compared to NO alone. For most of the transcripts downregulated by NO, 9 genes (90%) did not show changes in their expression levels in the presence of hypoxia compared to their expression with NO alone. Interestingly Fxyd5, which was the most prominent downregulated transcript by NO (18-folds) was significantly relieved of this effect when subjected to hypoxia plus NO.

**Table 3:** Impact of hypoxia on genes regulated by NO.

**Symbol**	**Gene Name**	**Ac**	**NO**	**Hyp + NO**
Ddit4	DNA-damage-inducible transcript 4	NM_029083.1	5.19	35.21

Trib3	tribbles homolog 3 (Drosophila)	NM_144554.1	5.85	27.11

Bnip3	BCL2/adenovirus E1B 19 kDa-interacting protein 1, NIP3	NM_009760.2	2.53	20.06

Phlda3	pleckstrin homology-like domain, family A, member 3	NM_013750.1	3.88	9.41

Nqo1	NAD(P)H dehydrogenase, quinone 1	U12961.1	2.99	8.32

Atf3	activating transcription factor 3	NM_007498.2	2.37	7.26

Vegfa	vascular endothelial growth factor A	NM_009505.2	2.06	6.55

Sesn2	sestrin 2	NM_144907.1	3.47	5.23

Adh7	alcohol dehydrogenase 7 (class IV), mu or sigma polypeptide	NM_009626.2	2.71	5.06

Gpt2	glutamic pyruvate transaminase (alanine aminotransferase) 2	NM_173866.1	2.49	5.02

Slc7a11	solute carrier family 7 (cationic amino acid transporter, y+ system), member 11	NM_011990.1	2.68	4.74

Cdkn1a	cyclin-dependent kinase inhibitor 1A (P21)	NM_007669.2	2.07	4.25

Scd1	stearoyl-Coenzyme A desaturase 1	NM_009127.2	4.18	3.55

Sfrs1	splicing factor, arginine/serine-rich 1 (ASF/SF2)	NM_173374.2	2.82	3.42

Slc19a2	solute carrier family 19 (thiamine transporter), member 2	NM_054087.1	2.32	3.08

Atp6ap2	ATPase, H+ transporting, lysosomal accessory protein 2	NM_027439.2	2.31	2.62

Txndc1	thioredoxin domain containing 1	NM_028339.1	2.03	2.58

Adam10	a disintegrin and metalloprotease domain 10	NM_007399.1	2.07	2.55

Lss	lanosterol synthase	AK012813.1	2.12	2.48

Pmaip1	phorbol-12-myristate-13-acetate-induced protein 1	NM_021451.1	2.27	2.46

Eif4g2	eukaryotic translation initiation factor 4, gamma 2	NM_013507.2	2.16	2.45

Sgpp1	sphingosine-1-phosphate phosphatase 1	NM_030750.2	3.00	2.42

Rbl2	retinoblastoma-like 2	NM_011250.2	2.01	2.33

D4Ertd765e	DNA segment, Chr 4, ERATO Doi 765, expressed	NM_026728.1	2.91	2.30

Vcl	vinculin	NM_009502.3	2.02	2.22

Pabpc1	poly A binding protein, cytoplasmic 1	NM_008774.2	2.11	2.19

Golph3	golgi phosphoprotein 3	NM_025673.2	2.18	2.15

Rrm1	ribonucleotide reductase M1	NM_009103.2	2.97	2.11

Nsmaf	neutral sphingomyelinase (N-SMase) activation associated factor	NM_010945.1	2.43	2.05

Dirc2	disrupted in renal carcinoma 2 (human)	NM_153550.2	2.23	2.03

Crip1	cysteine-rich protein 1 (intestinal)	NM_007763.1	-2.13	-2.09

Trerf1	transcriptional regulating factor 1	NM_172622.1	-2.14	-2.12

Rps19	ribosomal protein S19	NM_023133.1	-2.04	-2.18

Son	Son cell proliferation protein	NM_178880.3	-2.06	-2.23

Idb1	inhibitor of DNA binding 1	NM_010495.1	-2.45	-2.36

Nnt	nicotinamide nucleotide transhydrogenase	AK087064.1	-2.04	-2.41

Dhrs6	dehydrogenase/reductase (SDR family) member 6	NM_027208.1	-2.60	-2.42

Lst1	leukocyte specific transcript 1	NM_010734.1	-2.26	-2.55

Hps6	Hermansky-Pudlak syndrome 6	NM_176785.1	-4.86	-4.21

Fxyd5	FXYD domain-containing ion transport regulator 5	BC031112.1	-18.27	-9.71

This list of 40 genes was obtained by comparing the NO (0.5 mM DETA-NO)-generated gene profile to that generated by hypoxia (1% O_2_) plus NO. Hyp = hypoxia treatment; NO = nitric oxide treatment.

In another set of data analysis we determined the effect of NO on the gene profiles of hypoxia-generated transcripts. A comparison of gene profiles generated by hypoxia was performed against the profile generated by cells treated with hypoxia plus NO [Additional file 4]. A total of 100 transcripts were obtained in this category, with 81 genes being upregulated, while 20 were downregulated by both conditions (hypoxia vs. or hypoxia plus NO). Fifty-five transcripts upregulated under both conditions did not show significant changes in expression profiles when NO was added compared to hypoxia alone, whereas 26 genes in this category (32.1%) were influenced by NO. Interestingly, amongst the 26 genes that showed changes in gene expression due to the presence of NO, the majority of 17 genes (65.4%) showed a lower level of expression when cells were cotreated with hypoxia and NO compared to hypoxia alone. This suggests that NO attenuated the induction of hypoxia-induced transcripts. One plausible explanation for this observation could be attributed to the ability of NO to destabilize HIF-1α under hypoxic condition. Further studies involving HIF-1α deficient cells are necessary to support the role of HIF-1α in this process. On the other hand, NO was able to augment the expression of a few transcripts (9 genes, 34.6%) upregulated by hypoxia alone. The heterogeneous influence of NO on hypoxia-regulated gene profiles is in agreement with its ability to participate in direct and indirect signaling and to induce overlapping signaling pathways with hypoxia. NO activates soluble guanylyl cyclase to generate cGMP and in turn to activate cGMP-dependent protein kinases [40]. NO also reacts with superoxide (O_2_^-^) to generate peroxynitrite (ONOO^-^), potentially causing tissue damage and nucleic acid modifications [40,41]. There is evidence that large amounts of NO and ROS, produced during inflammation shift the NO chemistry towards indirect effects such as nitrosation, nitration and oxidation [42].

Finally, we compared the array data from all treatments to identify transcripts that were only seen in cells cotreated with hypoxia and NO but neither with hypoxia or NO alone. One-hundred and sixty-two unique transcripts were identified in this category, suggesting that the cross-talk between hypoxia and NO might be responsible for their regulation. The majority of the transcripts (122 genes, 75.9%) were upregulated, while the other 40 transcripts (24.1%) were downregulated [Additional file 5]. A few of the genes identified here had been reported by other investigators to be regulated by NO (e.g., SparC,) or hypoxia (e.g. Clk, Myd116, Ceacam1) but not by the combination of both. The majority of the unique transcripts identified in this study are unknown to be hypoxia or NO-dependent. This finding suggests alternative pathways for the regulation of these unique transcripts, which may involve multiple scenarios of cross-talks between hypoxia and NO operating in inflammation or cancer. Taken together, from our analysis and gene expression profiles generated in many array studies with different cell types, one can conclude that there is a core of genes consistently modulated by hypoxia or NO, while a large number of genes exhibited cell-type-specific changes.

### Verification of micro array data by qRT-PCR analysis

Despite the fact that our microarray data can be correlated to previous array studies with similar regulation patterns for several gene transcripts, we performed an independent validation of our data by RT-PCR after the isolation of whole cell RNA either from RAW 264.7 or primary mouse macrophages. Nine genes (ATF3, Ddit4, Sesn2, Vegf1, Trib3, Cdkn1a, Bnip3, Frat2 and Scd1) that were either up- or downregulated in our as well as other arrays, were chosen. Result obtained by RT-PCR (Table 4) confirmed the regulation pattern of the selected genes obtained by microarray analysis. However, there were gene specific variations in the expression levels for some of the transcripts. Changes in gene expression levels obtained with RT-PCR tend to be slightly lower compared to microarray data for most of the genes examined. Similar observations have been reported by other studies [43] and were attributed to different factors, including the sensitivity of the different methods, cross-interference between transcripts because of the large size of transcripts examined together (multiplexing) and the quality of mRNA [44].

**Table 4:** RT-PCR analysis of genes selected form the microarray profile.

		**RAW**		**Primary Macrophages**	
Symbol	Ac	Hyp	NO	Hyp	NO

Vegf1	NM_009505.2	5.7 ± 0.27	2.0 ± 0.14	5.5 ± 0.84	3.0 ± 0.46

Ddit4	NM_029083.1	16.6 ± 0.58	3.6 ± 0.53	2.4 ± 0.37	1.4 ± 0.30

Sesn2	NM_144907.1	2.0 ± 0.47	4.2 ± 0.33	2.5 ± 0.64	3.6 ± 0.70

Bnip3	NM_009760.2	24.1 ± 0.42	2.3 ± 0.40	3.6 ± 0.57	2.2 ± 0.56

Trib3	NM_144554.1	3.0 ± 0.51	3.4 ± 0.61	1.5 ± 0.32	1.7 ± 0.35

Atf3	NM_007498.2	4.6 ± 0.54	1.3 ± 0.27	0.9 ± 0.35	0.9 ± 0.31

Frat2	NM_177603.1	2.0 ± 0.45	1.3 ± 0.43	2.1 ± 0.63	1.6 ± 0.51

Cdkn1a	NM_007669.2	3.6 ± 0.61	2.3 ± 0.28	5.3 ± 1.33	4.2 ± 1.30

Scd1	NM_009127.2	1.3 ± 0.20	0.9 ± 0.60	1.0 ± 0.30	1.4 ± 0.54

RAW cells were subjected to hypoxia (1% O_2_) or NO (0.5 mM DETA-NO) for 6 h and primary macrophages for 12 h. Whole cell mRNA was isolated and analyzed by qRT-PCR. Expression changes of 9 genes were evaluated in relation to the values obtained in parallel for 16S ribosomal protein obtained from 4 individual experiments. Hyp = hypoxic treatment; NO = nitric oxide treatment.

### Proteomics analysis

**Figure 2:**
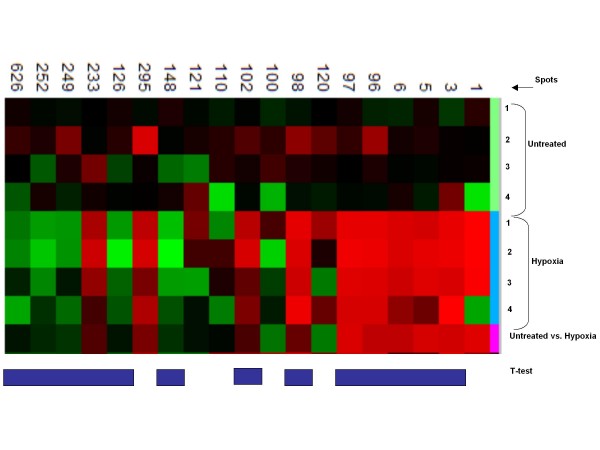
**Proteomics data of macrophages subjected to hypoxia for 6 hour vs. untreated cells**. The scanned gel contains four samples for each treatment (untreated or hypoxia (1% O_2_) treated) and a column comparing the median intensities of the same spot for the four samples in each treatment. Spot colors indicate: upregulation (red), downregulation (green), no change in regulation (dark/black). Student t-test for significantly regulated spots (< 0.05) is indicated by blue vertical bars.

A number of studies addressed the question whether changes in mRNA are reflected in corresponding changes in protein abundance [41,45-47]. Often, poor correlations have been reported between RNA transcriptions and protein levels and in some cases, little or no correlations were obtained [46]. Given the complexities of protein expression and the many stages at which it may be controlled, differences between RNA expression and protein expression are perhaps to be expected. Nevertheless we performed 2-D gel protein analysis to examine a genome wide change in protein expression of RAW cells after hypoxia- and NO-treatments to verify array data. Sample aliquots were labeled with two different fluorescent dyes (Cy3 and Cy5) and the internal control standard, actin, was stained with Cy2. 2-D PAGE was prepared and run as previously described [48]. Gels were scanned and data analysis was preformed by the Bioinformatic Section of Genedata, Basel, Switzerland. Comparing the intensity of the fluorescence of the scanned gels containing four samples for each treatment (untreated or hypoxia treated), we identified 19 spots that showed at least twofold higher or lower changes in protein expression compared to controls (Figure 2). Expression changes in 13 (all 19 except spots 1, 120,100,110,121 and 295) spots, regulated after hypoxia, were significant as estimated by the student T-test (< 0.05). Eight (61.5%) of the 13 significantly regulated spots were upregulated, whereas 5 (38.5%) were downregulated. Interestingly, this finding correlates with our array studies, where hypoxia upregulated more gene transcripts than it downregulated.

**Figure 3:**
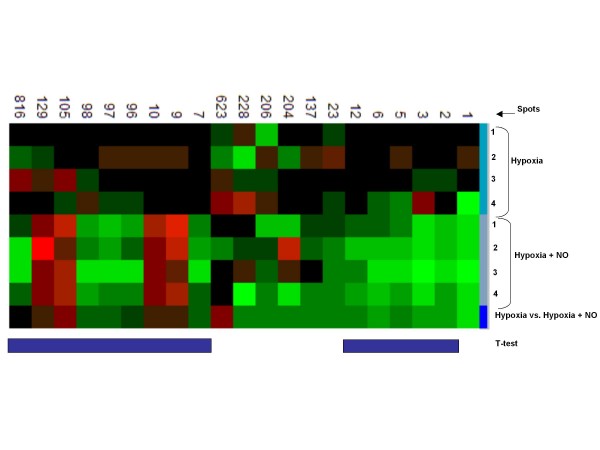
**Proteomics data comparing effects of nitric oxide on hypoxia regulated spots**. RAW cells were subjected for 6 hours to hypoxia (1% O_2_) or hypoxia plus NO (0.5 mM DETA-NO). The scanned gel contains four samples for each treatment (hypoxia or hypoxia plus NO) and a column comparing the median intensities of the same spot for the four samples in each treatment. Spot colors indicate: upregulation (red), downregulation (green), no change in regulation (dark/black). Student t-test for significantly regulated spots (< 0.05) is indicated by blue vertical bars.

To analyze the effect of NO on hypoxia regulated spots, we compared the expression patterns of hypoxia vs. hypoxia plus NO data (Figure 3). Twenty-one spots showed at least +2 or -2 folds changes in protein expression when cells were treated with hypoxia alone compared to cells treated with a combination of hypoxia and NO. Thirteen of the regulated proteins were significant by student T-test. In this analysis, the majority, 9 spots (69.2%) out of the 13 significant spots were downregulated and only 4 spots (9, 10,105 and 125) were up regulated. The higher number of downregulated spots seen when cells were cotreated with hypoxia and NO is consistent with the array data and suggest that NO predominantly attenuates gene expression when combined with hypoxia. Furthermore, spots 2, 5, 6, 96, and 98 of figure 2, which were significantly upregulated by hypoxia, were downregulated when macrophages were cotreated with hypoxia and NO (Figure 3). There was a strong correlation between the array and the proteomics data based on regulatory patterns seen on gene regulation profiles as a whole as well as on the effect of NO on hypoxia regulated gene patterns. However, at the level of individual proteins, we were unable to prove corresponding gene regulation patterns for specific genes/proteins. Finally to identify and annotate some of the differentially expressed spots of interest, we conducted mass spectrometry (MS) analysis. A total of 11 spots significantly regulated by hypoxia and/or NO were chosen for further identification. We identified spots 2, 6, 96, 97 and 98, which were significantly upregulated by hypoxia in this study as cofilin-1 (P45594), ferretin heavy chain (P09528, P02794), comm domain-containing protein7 (Q8BG94), ferritin light chain-1 protein (P15532) and peroxiredoxin (P35700), respectively. Consistent with our studies, antioxidants (peroxiredoxins), iron homeostatic (ferritin) and cofilin-1 are implicated in the hypoxia signaling cascade [48-51]. Interestingly, we are reporting for the first time the identification of comm domain containing protein 7 as significantly upregulated in cells treated with hypoxia. Presumably all COMM_Domain containing proteins are located in the nucleus and the COMM domain plays a role in protein-protein interactions. Several family members have been shown to bind and inhibit NF-κB. Other proteins identified in this study include nucleoside diphosphate kinase B (spot 7, P15532) and 40S ribosomal protein S5 (P97461), which were significantly downregulated when macrophages were cotreated with hypoxia and NO but not by hypoxia alone. The interplay between NO and hypoxia has important functional implications, expanding and enriching the possibilities for modulating stress responses.

## Conclusion

By eliminating the interference of steady state mRNA in gene expression profiling, we obtained lower number of significantly regulated transcripts in our study compared to published microarray data and identified previously unknown hypoxia-induced targets. Gene analysis profiling corroborated the interplay between NO- and hypoxia-induced signaling.

## Methods

### Cell culture

RAW 264.7 cells were cultured in RPMI 1640 supplemented with 2 mM glutamine, 100 units/ml penicillin, 100 μg/ml streptomycin and 10% fetal calf serum. Primary peritoneal mouse macrophages were isolated by peritoneal lavage with 15 ml PBS, supplemented with 10% fetal calf serum and cultured in RPMI 1640 with supplements as described. Cells were kept at 37°C in a humidified atmosphere with 5% CO_2_.

### Nuclei isolation

6 × 10^6 ^RAW 264.7 cells were seeded in 10-cm dishes one day prior to experiments. The following day, medium was changed, cells were treated as indicated and harvested. Nuclei were prepared in 200 μl hypotonic lysis buffer (10 mM Hepes, 0.1 mM EDTA, 2 mM MgCl_2_, 10 mM KCl, protease inhibitor cocktail, 1 mM DTT, 5 mM PMSF, pH 7.9) and incubated on ice for 10 min. After centrifugation (500 × g, 30 min, 4°C), the supernatant was discarded and nuclei were resuspended in 150 μl nuclei storage buffer (50 mM Tris-HCl, 0.1 mM EDTA, 5 mM MgCl_2_, and 40% glycerol, pH 8.3). Stalled transcriptions were allowed to resume following exogenous addition of nuclear run-on reaction buffer and reaction proceeded for 30 min at 30°C. Nuclear RNA was isolated, extracted and cDNA was hybridized to a mouse genomic array chip containing 32000 genes as previously described [26].

### Nuclear run-on

1 × 10^7 ^nuclei in 150 μl storage buffer were added to 150 μl nuclear run-on reaction buffer (10 mM Tris-HCl, 5 mM MgCl_2_, 300 mM KCl, 1 mM ATP, 1 mM GTP, 1 mM CTP, 1 mM UTP, pH 8.0) and nuclear run-on was performed for 30 min at 30°C. Nuclear RNA was isolated using the peqGOLD RNAPure kit according to manufacture's instructions (Peqlab, Erlangen, Germany).

### Microarray analysis

Identification of differentially expressed genes between normoxic and hypoxic treated NPC cells was carried out using GeneChip_Mous Genome Arrays (Affymetrix, Santa Clara, CA), which represented 32000 transcripts. In brief, 10 μg of total RNA was used to synthesize cDNA. The cDNA was then used as a template to generate biotinylated cRNA by in vitro transcription. The cRNA was subsequently fragmented. The quality and size distribution of total RNA, cRNA and fragmented cRNA were checked on an Agilent 2100 Bioanalyzer (Agilent, Amstelveen, The Netherlands), using RNA 6000 Pico assay. The fragmented cRNA was hybridized to the GeneChip arrays. The arrays were then washed, stained and finally scanned with a laser scanner. The scanned images were processed with GeneChip_Microarray Suite 5.0 (Affymetrix). Comparison between expression profiles of the normoxic and hypoxic and/or NO were performed using GeneSpring_software version 7 (Silcongenetics, Redwood, California). Gene expression data were first normalized by GC-RMA (robust multichip average) preprocessor. Normalized values below 0.001 were set to 0.001. The normalized expression values were then compared between normoxic and hypoxic, normoxic and nitric oxide, as well as for normoxia and hypoxia plus nitric oxide. Fold change differences were calculated to identify the up- and down-regulated genes. Statistical algorithms implemented in the Affymetrix microarray analysis software were applied to identify genes whose overall expression level in three independent experiments was altered at least 2-fold up or down by hypoxia and/or NO compared to untreated controls. To compare the gene profiles of the different treatments and to determine the number of transcripts regulated by each treatment, we eliminated duplicates of transcripts represented more than once as well as transcripts with unknown gene identification numbers.

### Quantitative real time RT-PCR

RT-PCR analyses were performed for a selection of upregulated and downregulated genes to confirm their differential expressions. 2 × 10^6 ^RAW 264.7 cells and/or primary peritoneal macrophages were seeded in 10-cm dishes one day prior to experiments. The following day medium was changed and cells were treated as indicated. Total RNA was isolated using the peqGOLD RNAPure kit (Peqlab, Erlangen, Germany). The reverse transcription was completed from 1 μg RNA with a IScript™ cDNA Synthesis RT-PCR kit. The quantitative real time PCR was performed by MyiQ (Bio-Rad). Reaction mixtures containing SYBR Green were composed according to the manufacturer protocol. The cycling program was: 50°C, 2 minutes; 95°C, 15 minutes; followed by 35 cycles at 95°C, 15 seconds; 60°C, 30 seconds; 72°C, 30 seconds. Values of Atf3, Ddit4, Vegf1, Bnip3, Sesn2, Cdkn1a and Frat2 were then normalized to 16S ribosomal protein. The following primer pairs were selected for quantitative real time PCR: Vegf1 forward: 5'-CAGGCTGCTGTAACGATGAA-3', Vegf1 reverse: 5'-GCATTCACATCTG-CTGTGCT-3'; Bnip3 forward: 5'-GGTTTTCCCCAAAGGAATA-3', Bnip3 reverse: 5'-TGACCACC-CAAGGTAATGGT-3'; Atf3 forward: 5'-CAGAGCCTGGTGTTGTGCTA-3,' Atf3 reverse: 5'-GGTGTCGTCCATCCTCTGTT-3'; Ddit4 forward: 5'-T-TCATTCGGA-TAGCAG-3', Ddit4 reverse: 5'-TCAGGTTGGCCAGGTG-3'; Sesn2 forward: 5'-GCATT-ACCTGCTGCTGCATA-3', Sesn2 reverse: 5'-AAGGCCTGGATATGCTCCTT-3'; Frat2 forward: 5'-GAATCGGGAGGGCTTCTAAC-3', Frat2 reverse: 5'-GCTCT-GCAATTGTA-GCACCA-3'; Cdkn1a forward: 5'-GGGATGGCAGTTAGGACTCA-3', Cdkn1a reverse: 5'-GTGGGGC-AAGTGCCTAGATA-3'; 16S ribosomal protein forward: 5'-AGATGATCGAGCCGCGC-3'; 16S ribosomal protein reverse: 5'-GCTACCAGGGCCTTTGAGATGGA-3'

### 2D DIGE gel analysis

Cells were washed twice in PBS, pelleted by centrifugation, and lyzed in lysis buffer (7 M urea, 2 M thiourea, 4% w/v CHAPS, 30 mM Tris-HCl, pH 8.5) by sonication. Insoluble material was removed by centrifugation at 12000 g for 5 min at 4°C. Precipitation of proteins was performed using the 2D clean-up kit (GE healthcare) according to manufacture guidelines and protein concentration was determined using the RC/DC protein assay (Bio-Rad). 50 μg of each lysate was labeled with Cy3 and Cy5 (400 pmol) in the dark for 30 min and quenched with 50-fold molar excess of free lysine-to-dye. Samples were reverse-labeled in order to enable all comparisons and eliminate any dye-labeling bias. Additionally samples were mixed and run on the same gels with an equal amount (50 μg) of Cy2-labeled standard. Cy2 was used as a standard on all gels to aid image matching and cross-gel statistical analysis.

Isoelectric focusing (IEF) was performed with IPGphor apparatus (GE Healthcare) according to manufacturer's instruction. Briefly, immobilized 24 cm linear pH gradient (IPG) strips, pH 3-10, were rehydrated (30 mM Tris Base,7 M urea, 2 M thiourea, 4% CHAPS, 0.5% IPG buffer, 50 mM DTT PH 8,5) overnight. The strips were focused according to the following protocol: linear ramp to 1000 V over 2 h, linear ramp to 4000 V over 1 h, linear ramp to 8000 V over 1 h and 8000 V for 68 kVh. Strips were equilibrated for 15 min in equilibration buffer (1.5 M TrisCl, pH 8.8, 6 M urea, 30% (v/v) glycerol, 2% (w/v) SDS, and trace amounts of bromphenol blue) containing 1% (w/v) DTT and thereafter in SDS Equilibration buffer containing 4% (w/v) iodoacetamide for 15 additional min. After equilibration, proteins were separated on 12% polyacrylamide gels using an Ettan Dalt Six device (GE Healthcare) at 22°C. Gels were run at 2.5 W/gel for 30 min and then 15 W/gel until the tracking dye had migrated off the bottom of the gel.

### Image analysis

Gels were scanned with a Typhoon 9410 variable mode imager (Amersham Biosciences) using excitation/emission wavelengths specific for Cy2 (488 nm/520 nm), Cy3 (532 nm/580 nm), and Cy5 (633 nm/670 nm). Images were normalized, statistically analyzed, and differentially expressed proteins were identified and quantified using DeCyder (GE Healthcare). MS identification was carried out for proteins that showed more than two-fold variation in abundance. Briefly, the protein spots were cut out of 2-D gels using Gelpix Spot-Excision Robot (Genetix, Hampshire, UK) and then washed three times with Milli-Q water. According to the manufacture of ZipPlate micro-SPE Plate, gel pieces were transferred into ZipPlate micro-SPE Plate wells (Millipore, Billerica, MA, USA). The gel pieces were dried in a vacuum and the proteins were digested overnight in 10 ml trypsin (10 ng/ml, Trypsin Gold, mass spectrometry grade, Promega, Madison, WI, USA) in 25 mM ammonium bicarbonate at 37°C. Peptide fragments were extracted with 0.2% TFA for 30 min, applied onto the C18 resin, and then desalted with 0.2% TFA. The tryptic peptide mixtures were recovered with 5 ml elution solution containing 50% ACN/0.1% TFA by centrifugation for 15 s at 17506 g and spotted onto a MALDI sample target plate. Peptide mass spectra were obtained on a MALDI-TOF/TOF mass spectrometer (4700 Proteomics Analyzer, Applied Biosystems, Foster City, CA, USA).

### Database search

Protein identification was processed and analyzed by searching the Swiss-Prot and NCBI protein database using the MASCOT search engine of Matrix Science, integrated in the Global Protein Server (GPS) Workstation. The mass tolerance, the most important parameter, was limited to 50 ppm. The results from the MS and MS/MS spectra were accepted as a good identification when the GPS score confidence was higher than 95%.

### Statistical analysis

The nuclear run-on data was statistically analyzed by Genedata AG (Basel, Switzerland). The p-value cut-off was set to 0.0001 or 0.001 and the expected number of transcripts with p-values below these thresholds are about 15 or 150, respectively, assuming random effects only (33155 transcripts on the microarray, thereof ~15000 with valid values). False discovery rates (FDR) were estimated according to a balanced permutation test with 500 repeats. In addition the ratios of the groups means (N-fold changes) were determined. The significant transcripts were in addition determined on the condition that at least 2 out of 3 experiments have valid values in each group. Representative data are shown. For the quantitative real time verification of microarray data, gene expression was shown as mean values ± standard deviation (SD). Means were checked for statistical significance using 1-way analysis of variance (ANOVA), followed by Tukey tests. Differences having a P value < 0.05 were considered to be statistically significant.

## Authors' contributions

EII and BB proposed, designed, analyzed and interpreted data. EII, ND and BB prepared the manuscript. NA performed the array and proteomics studies. SE carried out quantitative RT-PCR studies.

## Supplementary Material

Additional file 1 **Transcripts regulated after exposing cells to hypoxia (1% O_2_) for 6 h**. Ac, indicates the gene accession number. Significantly regulated are those transcripts induced or repressed ≥ 2-folds vs. controls. Click here for file

Additional file 2 **Transcripts regulated after exposing cells to 0.5 mM DETA-NO for 6 h**. Ac, indicates the gene accession number. Significantly regulated are those transcripts induced or repressed ≥ 2-folds vs. controls. Click here for file

Additional file 3 **Transcripts regulated after exposing cells to a combination of hypoxia and NO**. Ac, indicates the gene accession number. Significantly regulated are those transcripts induced or repressed ≥ 2-folds vs. controls following the exposure to hypoxia (1% O_2_) plus 0.5 mM DETA-NO for 6 h. Click here for file

Additional file 4 **Impact of NO on genes regulated by hypoxia**. This list compared gene profiles generated by vs. that generated by hypoxia and NO. Hyp = hypoxic treatment (1% O_2_); NO = nitric oxide treatment (0.5 mM DETA-NO). Click here for file

Additional file 5 **Transcripts unique to the treatment of cells with the combination of hypoxia and NO**. The cross-talk between hypoxia and NO generated 162 transcripts unique to treatment of cells with the combination of hypoxia and NO. Hyp = hypoxic treatment (1% O_2_); NO = nitric oxide treatment (0.5 mM DETA-NO). Click here for file
