# Supplementary material for: Hypoxic transcription gene profiles under the modulation of nitric oxide in nuclear run on-microarray and proteomics
Source: BMC Genomics. 2009 Sep 2;10:408. doi: 10.1186/1471-2164-10-408 (PMC2743718; doi:10.1186/1471-2164-10-408)
Supplement: Additional file 1 — Transcripts regulated after exposing cells to hypoxia (1% O2) for 6 h. Ac, indicates the gene accession number. Significantly regulated are those transcripts induced or repressed ≥ 2-folds vs. controls. [file 1471-2164-10-408-S1.doc]

**Suppl. Table 1 - Transcripts regulated after exposing cells to hypoxia (1% O_2_) for 6 h**

| **UP** | **Gene name** | **Ac** | **Folds** |
| --- | --- | --- | --- |
| Bnip3 | BCL2/adenovirus E1B 19kDa-interacting protein 1, NIP3 | NM_009760.2 | 33.40 |
| Selenbp2 : Selenbp1 | selenium binding protein 2 : selenium binding protein 1 | NM_009150.2 | 23.96 |
| Ddit4 | DNA-damage-inducible transcript 4 | NM_029083.1 | 19.68 |
| Ndrg1 : Ndrl | N-myc downstream regulated gene 1 : N-myc downstream regulated-like | NM_010884.1 | 13.18 |
| Vegfa | vascular endothelial growth factor A | NM_009505.2 | 9.08 |
| Fbxo23 | F-box only protein 23 | NM_028841.1 | 8.09 |
| Serpine1 | serine (or cysteine) proteinase inhibitor, clade E, member 1 | NM_008871.1 | 8.06 |
| Trib3 | tribbles homolog 3 (Drosophila) | NM_144554.1 | 8.05 |
| Atf3 | activating transcription factor 3 | NM_007498.2 | 7.27 |
| Ier3 | immediate early response 3 | NM_133662.1 | 7.21 |
| F10 | coagulation factor X | NM_007972.2 | 7.06 |
| Gys3 : Gys1 | glycogen synthase 3, brain : glycogen synthase 1, muscle | NM_030678.2 | 6.30 |
| Pgm2 | phosphoglucomutase 2 | NM_028132.2 | 6.24 |
| Olfr39 | olfactory receptor 39 | NM_146825.1 | 6.22 |
| Hmox1 | heme oxygenase (decycling) 1 | NM_010442.1 | 6.14 |
| Rcor1 | RE1-silencing transcription factor (REST) co-repressor 1 | NM_054048.1 | 5.69 |
| Oasl2 | 2'-5' oligoadenylate synthetase-like 2 | NM_011854.1 | 5.35 |
| Hyal1 | hyaluronidase 1 | NM_008317.2 | 5.09 |
| Egln1 | EGL nine homolog 1 (C. elegans) | NM_053207.1 | 4.99 |
| Pfkl | phosphofructokinase, liver, B-type | AK036318.1 | 4.99 |
| Grhpr | glyoxylate reductase/hydroxypyruvate reductase |  | 4.75 |
| P4ha1 | procollagen-proline, 2-oxoglutarate 4-dioxygenase (proline 4-hydroxylase), alpha 1 polypeptide |  | 4.52 |
| Ero1l | ERO1-like (S. cerevisiae) | NM_015774.2 | 4.28 |
| Plod1 | procollagen-lysine, 2-oxoglutarate 5-dioxygenase 1 | NM_011122.1 | 4.12 |
| Slc16a3 | solute carrier family 16 (monocarboxylic acid transporters), member 3 | NM_030696.2 | 4.11 |
| Cox6a2 | cytochrome c oxidase, subunit VI a, polypeptide 2 | BC028514.1 | 4.07 |
| Myl7 | myosin, light polypeptide 7, regulatory | NM_022879.1 | 3.93 |
| Rnf128 | ring finger protein 128 | NM_023270.3 | 3.86 |
| Nfil3 | nuclear factor, interleukin 3, regulated | NM_017373.2 | 3.79 |
| Pde4a | phosphodiesterase 4A, cAMP specific | NM_019798.2 | 3.76 |
| Amhr2 | anti-Mullerian hormone type 2 receptor | NM_144547.1 | 3.72 |
| Bnip3l | BCL2/adenovirus E1B 19kDa-interacting protein 3-like | NM_009761.2 | 3.72 |
| Stc2 | stanniocalcin 2 | AK002527.1 | 3.63 |
| Slc37a4 | solute carrier family 37 (glycerol-6-phosphate transporter), member 4 | NM_008063.1 | 3.55 |
| Jmjd2b | jumonji domain containing 2B | NM_172132.1 | 3.51 |
| Tob1 | transducer of ErbB-2.1 | NM_009427.1 | 3.50 |
| Olfr1214 | olfactory receptor 1214 | NM_146897.1 | 3.48 |
| Bhlhb2 | basic helix-loop-helix domain containing, class B2 | NM_011498.2 | 3.47 |
| Tyki | thymidylate kinase family LPS-inducible member | NM_020557.3 | 3.36 |
| Bnip3l | BCL2/adenovirus E1B 19kDa-interacting protein 3-like | NM_009761.2 | 3.33 |
| Dip3b | Dip3 beta | NM_145220.1 | 3.27 |
| Mx2 | myxovirus (influenza virus) resistance 2 | NM_013606.1 | 3.17 |
| Spag1 | sperm associated antigen 1 | NM_012031.1 | 3.15 |
| Fto | fatso | NM_011936.1 | 3.14 |
| Eno1 | enolase 1, alpha non-neuron | NM_023119.1 | 3.07 |
| Cysltr1 | cysteinyl leukotriene receptor 1 | NM_021476.2 | 3.05 |
| Lmtn | limitin | NM_197889.1 | 3.05 |
| Vamp2 | vesicle-associated membrane protein 2 | NM_009497.2 | 3.01 |
| Gdap10 | ganglioside-induced differentiation-associated-protein 10 | NM_010268.1 | 2.99 |
| Pkm2 | pyruvate kinase, muscle | AK083076.1 | 2.94 |
| Hk2 | hexokinase 2 | NM_013820.1 | 2.91 |
| Pgk1 | phosphoglycerate kinase 1 | NM_008828.1 | 2.89 |
| Cdkn1a | cyclin-dependent kinase inhibitor 1A (P21) | NM_007669.2 | 2.89 |
| Slc41a3 | solute carrier family 41, member 3 | NM_177460.3 | 2.86 |
| Rara | retinoic acid receptor, alpha | BC010216.1 | 2.85 |
| Efna1 | ephrin A1 | NM_010107.2 | 2.85 |
| Pgk1 | phosphoglycerate kinase 1 | NM_008828.1 | 2.81 |
| Stc1 | stanniocalcin 1 | NM_009285.2 | 2.80 |
| Clcn3 | chloride channel 3 | NM_173876.1 | 2.78 |
| Irf7 | interferon regulatory factor 7 | NM_016850.1 | 2.77 |
| Gm2a | GM2 ganglioside activator protein | NM_010299.2 | 2.75 |
| Tef | thyrotroph embryonic factor | NM_153484.1 | 2.74 |
| Avil | advillin | NM_009635.2 | 2.73 |
| Gpi1 | glucose phosphate isomerase 1 | NM_008155.1 | 2.72 |
| Smox | spermine oxidase | NM_145533.1 | 2.69 |
| Mt2 | metallothionein 2 |  | 2.69 |
| Pgam1 | phosphoglycerate mutase 1 | NM_023418.1 | 2.68 |
| B3gnt3 | UDP-GlcNAc betaGal beta-1,3-N-acetylglucosaminyltransferase 3 | NM_028189.2 | 2.67 |
| Acvrl1 | activin A receptor, type II-like 1 | NM_009612.1 | 2.65 |
| Pbef1 | pre-B-cell colony-enhancing factor 1 | NM_021524.1 | 2.64 |
| Tpi | triosephosphate isomerase | NM_009415.1 | 2.62 |
| Oas2 | 2'-5' oligoadenylate synthetase 2 | NM_145227.1 | 2.56 |
| Nr1d1 | nuclear receptor subfamily 1, group D, member 1 | NM_145434.1 | 2.55 |
| Scd1 | stearoyl-Coenzyme A desaturase 1 | NM_009127.2 | 2.54 |
| Anxa2 | annexin A2 | NM_007585.2 | 2.53 |
| Zfp99 | zinc finger protein 99 | NM_023322.1 | 2.53 |
| D4Ertd765e | DNA segment, Chr 4, ERATO Doi 765, expressed | NM_026728.1 | 2.52 |
| Me2 | malic enzyme 2, NAD(+)-dependent, mitochondrial | AK039906.1 | 2.51 |
| Sertad1 | SERTA domain containing 1 | NM_018820.3 | 2.51 |
| Frat2 | frequently rearranged in advanced T-cell lymphomas 2 | NM_177603.1 | 2.49 |
| Tiparp | TCDD-inducible poly(ADP-ribose) polymerase | NM_178892.3 | 2.48 |
| H28 | histocompatibility 28 | BC024930.1 | 2.47 |
| Pdxp | pyridoxal (pyridoxine, vitamin B6) phosphatase | NM_020271.2 | 2.46 |
| Hyal1 | hyaluronidase 1 | NM_008317.2 | 2.43 |
| Fosl2 | fos-like antigen 2 | NM_008037.3 | 2.42 |
| Il15 | interleukin 15 | NM_008357.1 | 2.41 |
| Prss16 | protease, serine, 16 (thymus) | NM_019429.1 | 2.40 |
| Zfp292 | zinc finger protein 292 | NM_013889.1 | 2.40 |
| BC050210 | cDNA sequence BC050210 | NM_201365.1 | 2.38 |
| Ncoa4 | nuclear receptor coactivator 4 | NM_019744.1 | 2.37 |
| Pecam | platelet/endothelial cell adhesion molecule | NM_008816.1 | 2.36 |
| Nxn | nucleoredoxin | NM_008750.2 | 2.35 |
| Sesn2 | sestrin 2 | NM_144907.1 | 2.32 |
| Scd3 : Scd2 | stearoyl-coenzyme A desaturase 3 : stearoyl-Coenzyme A desaturase 2 | NM_009128.1 | 2.31 |
| Oas1b | 2'-5' oligoadenylate synthetase 1B | NM_011853.1 | 2.31 |
| Hk1 | hexokinase 1 | NM_010438.1 | 2.30 |
| Ddr2 | discoidin domain receptor family, member 2 | NM_022563.1 | 2.29 |
| Samsn1 | SAM domain, SH3 domain and nuclear localisation signals, 1 | NM_023380.1 | 2.29 |
| Fbxo21 | F-box only protein 21 | NM_145564.2 | 2.29 |
| Cbs | cystathionine beta-synthase | NM_144855.1 | 2.28 |
| Car12 | carbonic anyhydrase 12 | NM_178396.3 | 2.28 |
| Leprel2 | leprecan-like 2 | NM_013534.3 | 2.24 |
| Pira3 | paired-Ig-like receptor A3 | NM_011090.1 | 2.23 |
| Aldo1 | aldolase 1, A isoform | NM_007438.2 | 2.22 |
| Eif2c4 | eukaryotic translation initiation factor 2C, 4 | NM_153177.1 | 2.21 |
| Aldo1 | aldolase 1, A isoform | NM_007438.2 | 2.20 |
| Dgat2 | diacylglycerol O-acyltransferase 2 | NM_026384.2 | 2.18 |
| Hsd3b7 | hydroxy-delta-5-steroid dehydrogenase, 3 beta- and steroid delta-isomerase 7 | NM_133943.1 | 2.18 |
| Phospho1 | phosphatase, orphan 1 | BC035943.1 | 2.17 |
| Tmem25 | transmembrane protein 25 | NM_027865.1 | 2.17 |
| Lair1 | leukocyte-associated Ig-like receptor 1 | NM_178611.2 | 2.17 |
| Rest | RE1-silencing transcription factor | BC023433.1 | 2.17 |
| Adfp | adipose differentiation related protein | NM_007408.2 | 2.16 |
| Pscdbp | pleckstrin homology, Sec7 and coiled-coil domains, binding protein | NM_139200.2 | 2.16 |
| Ltbp2 | latent transforming growth factor beta binding protein 2 | NM_013589.1 | 2.16 |
| Oit3 | oncoprotein induced transcript 3 | NM_010959.1 | 2.15 |
| Nxf7 | nuclear RNA export factor 7 | NM_130888.1 | 2.15 |
| Pias3 | protein inhibitor of activated STAT 3 | NM_018812.1 | 2.14 |
| Sap30 | sin3 associated polypeptide | NM_021788.1 | 2.14 |
| Mvd | mevalonate (diphospho) decarboxylase | NM_138656.1 | 2.13 |
| Zfp598 | zinc finger protein 598 | NM_183149.1 | 2.13 |
| Usp18 | ubiquitin specific protease 18 | NM_011909.1 | 2.13 |
| Fliih | flightless I homolog (Drosophila) | NM_022009.1 | 2.12 |
| Map2k1 | mitogen activated protein kinase kinase 1 | NM_008927.1 | 2.12 |
| Galnact2 | chondroitin sulfate GalNAcT-2 | NM_030165.2 | 2.10 |
| Pex11c | peroxisomal biogenesis factor 11c | NM_026951.1 | 2.10 |
| Uba52 | ubiquitin A-52 residue ribosomal protein fusion product 1 | NM_019883.1 | 2.10 |
| H2-Q7 : H2-Q8 | histocompatibility 2, Q region locus 7 : histocompatibility 2, Q region locus 8 | NM_023124.2 | 2.09 |
| Acy1 | aminoacylase 1 | NM_025371.1 | 2.09 |
| Coro2a | coronin, actin binding protein 2A | AK030971.1 | 2.09 |
| Pafah1b3 | platelet-activating factor acetylhydrolase, isoform 1b, alpha1 subunit | NM_008776.1 | 2.08 |
| Socs1 | suppressor of cytokine signaling 1 | NM_009896.1 | 2.08 |
| Rbbp6 : C030034J04Rik | retinoblastoma binding protein 6 : RIKEN cDNA C030034J04 gene | NM_011247.1 | 2.08 |
| Pira3 | paired-Ig-like receptor A3 | NM_011090.1 | 2.07 |
| Samd8 | sterile alpha motif domain containing 8 | NM_026283.1 | 2.06 |
| Immp2l | inner mitochondrial membrane peptidase 2-like (S. cerevisiae) | NM_053122.2 | 2.06 |
| Cnnm4 | cyclin M4 | NM_033570.1 | 2.06 |
| Uaca | uveal autoantigen with coiled-coil domains and ankyrin repeats | NM_028283.1 | 2.05 |
| P4hb | prolyl 4-hydroxylase, beta polypeptide | NM_011032.1 | 2.05 |
| Tubb4 | tubulin, beta 4 | NM_009451.3 | 2.05 |
| Adar | adenosine deaminase, RNA-specific | NM_019655.2 | 2.05 |
| Depdc5 | DEP domain containing 5 | NM_177786.2 | 2.04 |
| Oas1g : Oas1a | 2'-5' oligoadenylate synthetase 1G : 2'-5' oligoadenylate synthetase 1A | NM_011852.2 | 2.04 |
| Isgf3g | interferon dependent positive acting transcription factor 3 gamma | NM_008394.2 | 2.04 |
| Gsn | gelsolin | NM_146120.2 | 2.04 |
| Ndst1 | N-deacetylase/N-sulfotransferase (heparan glucosaminyl) 1 | NM_008306.2 | 2.03 |
| Gypc | glycophorin C | NM_027863.2 | 2.03 |
| Atrn | attractin | NM_009730.1 | 2.03 |
| Elmo3 | engulfment and cell motility 3, ced-12 homolog (C. elegans) | NM_172760.1 | 2.02 |
| Als2cr2 | amyotrophic lateral sclerosis 2 (juvenile) chromosome region, candidate 2 homolog (human) | NM_172656.3 | 2.02 |
| Btg2 | B-cell translocation gene 2, anti-proliferative | NM_007570.1 | 2.01 |
| Kcnc3 | potassium voltage gated channel, Shaw-related subfamily, member 3 | NM_008422.1 | 2.01 |
| Fcgrt | Fc receptor, IgG, alpha chain transporter | NM_010189.1 | 2.00 |

| **DOWN** | **Gene name** | **Ac** | **Folds** |
| --- | --- | --- | --- |
| Saa3 | serum amyloid A 3 | NM_011315.2 | -2.02 |
| Rps29 | ribosomal protein S29 | NM_009093.1 | -2.03 |
| Eif2c1 | eukaryotic translation initiation factor 2C, 1 | NM_153403.1 | -2.04 |
| Gmfg | glia maturation factor, gamma | BC011488.1 | -2.05 |
| Hp | haptoglobin | NM_017370.1 | -2.07 |
| Hist1h1b | histone 1, H1b | NM_020034.1 | -2.10 |
| Slc11a2 | solute carrier family 11 (proton-coupled divalent metal ion transporters), member 2 | AK049856.1 | -2.11 |
| Armet | arginine-rich, mutated in early stage tumors | NM_029103.1 | -2.12 |
| Tnf | tumor necrosis factor | NM_013693.1 | -2.13 |
| Ly6g6c | lymphocyte antigen 6 complex, locus G6C | NM_023463.2 | -2.14 |
| Cd52 | CD52 antigen | NM_013706.1 | -2.15 |
| Saa3 | serum amyloid A 3 | NM_011315.2 | -2.15 |
| Rasgrp2 | RAS, guanyl releasing protein 2 | NM_011242.1 | -2.16 |
| Son | Son cell proliferation protein | NM_178880.3 | -2.16 |
| Wdr5b | WD repeat domain 5B | NM_027113.2 | -2.18 |
| Apoc2 | apolipoprotein C-II | NM_009695.2 | -2.19 |
| Mrpl52 | mitochondrial ribosomal protein L52 | NM_026851.1 | -2.21 |
| Ddx18 | DEAD (Asp-Glu-Ala-Asp) box polypeptide 18 | NM_025860.2 | -2.22 |
| Ndufa7 | NADH dehydrogenase (ubiquinone) 1 alpha subcomplex, 7 (B14.5a) | BC044054.1 | -2.22 |
| Mogat1 | monoacylglycerol O-acyltransferase 1 | NM_026713.1 | -2.28 |
| Mafb | v-maf musculoaponeurotic fibrosarcoma oncogene family, protein B (avian) | NM_010658.2 | -2.32 |
| Ovtn | oviductin | NM_172908.2 | -2.34 |
| Ikbke | inhibitor of kappaB kinase epsilon | NM_019777.2 | -2.37 |
| Nnt | nicotinamide nucleotide transhydrogenase | AK087064.1 | -2.39 |
| Ccl3 | chemokine (C-C motif) ligand 3 | X12531.1 | -2.42 |
| Trim17 | tripartite motif protein 17 | NM_031172.1 | -2.44 |
| Hist1h2ab : Hist1h2ad | histone 1, H2ab : histone 1, H2ad | NM_178188.1 | -2.47 |
| Ccl2 | chemokine (C-C motif) ligand 2 | NM_011333.1 | -2.58 |
| Clecsf12 | C-type (calcium dependent, carbohydrate recognition domain) lectin, superfamily member 12 | NM_020008.1 | -2.61 |
| Lst1 | leukocyte specific transcript 1 | NM_010734.1 | -2.62 |
| Hist1h2ao | histone 1, H2ao | NM_178185.1 | -2.67 |
| Hist1h2af : Hist1h2ae | histone 1, H2af : histone 1, H2ae | NM_175661.1 | -2.67 |
| Hist1h2ak | histone 1, H2ak | NM_178183.1 | -2.68 |
| Hist1h2an | histone 1, H2an | NM_178184.1 | -2.71 |
| Hist2h2aa1 : Hist2h2ac : Hist2h2ab | histone 2, H2aa1 : histone 2, H2ac : histone 2, H2ab | NM_178213.2 | -2.73 |
| Bcl2a1c | B-cell leukemia/lymphoma 2 related protein A1c | NM_007535.1 | -2.91 |
| Centa2 | centaurin, alpha 2 | NM_172133.1 | -2.95 |
| Chst1 | carbohydrate (keratan sulfate Gal-6) sulfotransferase 1 | NM_023850.1 | -3.10 |
| Bcl2a1d : Bcl2a1b : Bcl2a1a | B-cell leukemia/lymphoma 2 related protein A1d : B-cell leukemia/lymphoma 2 related protein A1b : B-cell leukemia/lymphoma 2 related protein A1a | NM_007536.1 | -3.22 |
| Egr1 | early growth response 1 | NM_007913.2 | -3.27 |
| Hps6 | Hermansky-Pudlak syndrome 6 | NM_176785.1 | -3.74 |
| Tfrc | transferrin receptor | NM_011638.3 | -3.86 |
| Fxyd5 | FXYD domain-containing ion transport regulator 5 | BC031112.1 | -4.20 |

Ac, indicates the gene accession number. Significantly regulated are those transcripts induced or repressed ≥ 2-folds vs. controls.
