# Supplementary material for: Hypoxic transcription gene profiles under the modulation of nitric oxide in nuclear run on-microarray and proteomics
Source: BMC Genomics. 2009 Sep 2;10:408. doi: 10.1186/1471-2164-10-408 (PMC2743718; doi:10.1186/1471-2164-10-408)
Supplement: Additional file 2 — Transcripts regulated after exposing cells to 0.5 mM DETA-NO for 6 h. Ac, indicates the gene accession number. Significantly regulated are those transcripts induced or repressed ≥ 2-folds vs. controls. [file 1471-2164-10-408-S2.doc]

**Suppl. Table 2 - Transcripts regulated after exposing cells to 0.5 mM DETA-NO for 6 h**

| **UP** | **Gene name** | **Ac** | **Folds** |
| --- | --- | --- | --- |
| Trib3 | tribbles homolog 3 (Drosophila) | NM_144554.1 | 5.85 |
| Ddit4 | DNA-damage-inducible transcript 4 | NM_029083.1 | 5.19 |
| Scd1 | stearoyl-Coenzyme A desaturase 1 | NM_009127.2 | 4.18 |
| Phlda3 | pleckstrin homology-like domain, family A, member 3 | NM_013750.1 | 3.88 |
| Sesn2 | sestrin 2 | NM_144907.1 | 3.47 |
| Sgpp1 | sphingosine-1-phosphate phosphatase 1 | NM_030750.2 | 3.00 |
| Nqo1 | NAD(P)H dehydrogenase, quinone 1 | U12961.1 | 2.99 |
| Rrm1 | ribonucleotide reductase M1 | NM_009103.2 | 2.97 |
| D4Ertd765e | DNA segment, Chr 4, ERATO Doi 765, expressed | NM_026728.1 | 2.91 |
| Lace1 | lactation elevated 1 | NM_145743.1 | 2.89 |
| Sfrs1 | splicing factor, arginine/serine-rich 1 (ASF/SF2) | NM_173374.2 | 2.82 |
| Adh7 | alcohol dehydrogenase 7 (class IV), mu or sigma polypeptide | NM_009626.2 | 2.71 |
| Slc7a11 | solute carrier family 7 (cationic amino acid transporter, y+ system), member 11 | NM_011990.1 | 2.68 |
| Bnip3 | BCL2/adenovirus E1B 19kDa-interacting protein 1, NIP3 | NM_009760.2 | 2.53 |
| Gpt2 | glutamic pyruvate transaminase (alanine aminotransferase) 2 | NM_173866.1 | 2.49 |
| Nsmaf | neutral sphingomyelinase (N-SMase) activation associated factor | NM_010945.1 | 2.43 |
| P2ry12 | purinergic receptor P2Y, G-protein coupled 12 | NM_027571.2 | 2.40 |
| Atf3 | activating transcription factor 3 | NM_007498.2 | 2.37 |
| Slc19a2 | solute carrier family 19 (thiamine transporter), member 2 | NM_054087.1 | 2.32 |
| Atp6ap2 | ATPase, H+ transporting, lysosomal accessory protein 2 | NM_027439.2 | 2.31 |
| Pmaip1 | phorbol-12-myristate-13-acetate-induced protein 1 | NM_021451.1 | 2.27 |
| Dirc2 | disrupted in renal carcinoma 2 (human) | NM_153550.2 | 2.23 |
| Ccl3 | chemokine (C-C motif) ligand 3 | X12531.1 | 2.22 |
| Golph3 | golgi phosphoprotein 3 | NM_025673.2 | 2.18 |
| Slmap | sarcolemma associated protein | NM_032008.2 | 2.18 |
| Foxd3 | forkhead box D3 | NM_010425.1 | 2.17 |
| Eif4g2 | eukaryotic translation initiation factor 4, gamma 2 | NM_013507.2 | 2.16 |
| Frat2 | frequently rearranged in advanced T-cell lymphomas 2 | NM_177603.1 | 2.15 |
| Ivns1abp | influenza virus NS1A binding protein | NM_028582.2 | 2.13 |
| Igf2bp1 | insulin-like growth factor 2, binding protein 1 | NM_009951.2 | 2.12 |
| Lss | lanosterol synthase | AK012813.1 | 2.12 |
| Pabpc1 | poly A binding protein, cytoplasmic 1 | NM_008774.2 | 2.11 |
| Clpx | caseinolytic protease X (E.coli) | NM_011802.1 | 2.09 |
| Mpst | mercaptopyruvate sulfurtransferase | NM_138670.1 | 2.08 |
| Cdkn1a | cyclin-dependent kinase inhibitor 1A (P21) | NM_007669.2 | 2.07 |
| Adam10 | a disintegrin and metalloprotease domain 10 | NM_007399.1 | 2.07 |
| Prom2 | prominin 2 | NM_138750.1 | 2.07 |
| Prkcn | protein kinase C, nu | NM_029239.2 | 2.06 |
| Vegfa | vascular endothelial growth factor A | NM_009505.2 | 2.06 |
| Cebpz | CCAAT/enhancer binding protein zeta | NM_009882.1 | 2.05 |
| Txndc1 | thioredoxin domain containing 1 | NM_028339.1 | 2.03 |
| Ttk | Ttk protein kinase | NM_009445.1 | 2.03 |
| Vcl | vinculin | NM_009502.3 | 2.02 |
| Ext2 | exostoses (multiple) 2 | NM_010163.1 | 2.02 |
| Rbl2 | retinoblastoma-like 2 | NM_011250.2 | 2.01 |

| **DOWN** | **Gene name** | **Ac** | **Folds** |
| --- | --- | --- | --- |
| Rhoc | ras homolog gene family, member C | NM_007484.1 | -2.00 |
| Cnot3 | CCR4-NOT transcription complex, subunit 3 | NM_146176.1 | -2.00 |
| Il8rb | interleukin 8 receptor, beta | D17630.1 | -2.01 |
| Grin2b | glutamate receptor, ionotropic, NMDA2B (epsilon 2) | NM_008171.2 | -2.01 |
| Cd52 | CD52 antigen | NM_013706.1 | -2.02 |
| Nnt | nicotinamide nucleotide transhydrogenase | AK087064.1 | -2.04 |
| Rps19 | ribosomal protein S19 | NM_023133.1 | -2.04 |
| Rpl3l | ribosomal protein L3-like | NM_025425.1 | -2.05 |
| Srd5a2l2 | steroid 5 alpha-reductase 2-like 2 | NM_153801.1 | -2.05 |
| Son | Son cell proliferation protein | NM_178880.3 | -2.06 |
| Hyal3 | hyaluronidase 3 | NM_178020.2 | -2.08 |
| Stfa1 | stefin A1 | NM_001001332.1 | -2.10 |
| Atp6v1g1 | ATPase, H+ transporting, V1 subunit G isoform 1 | NM_024173.1 | -2.12 |
| Lgals4 | lectin, galactose binding, soluble 4 | NM_010706.1 | -2.12 |
| Crip1 | cysteine-rich protein 1 (intestinal) | NM_007763.1 | -2.13 |
| Trerf1 | transcriptional regulating factor 1 | NM_172622.1 | -2.14 |
| Madd | MAP-kinase activating death domain | NM_145527.2 | -2.16 |
| S100a11 | S100 calcium binding protein A11 (calizzarin) | NM_016740.2 | -2.16 |
| Olfr313 | olfactory receptor 313 | NM_146536.1 | -2.17 |
| Idb2 | inhibitor of DNA binding 2 | M69293.1 | -2.20 |
| Myb | myeloblastosis oncogene | NM_010848.2 | -2.24 |
| Fut2 | fucosyltransferase 2 | NM_018876.2 | -2.25 |
| Anapc13 | anaphase promoting complex subunit 13 | NM_181394.1 | -2.26 |
| Lst1 | leukocyte specific transcript 1 | NM_010734.1 | -2.26 |
| Kcnb1 | potassium voltage gated channel, Shab-related subfamily, member 1 | NM_008420.3 | -2.27 |
| Lgals7 | lectin, galactose binding, soluble 7 | NM_008496.3 | -2.30 |
| Rbm3 | RNA binding motif protein 3 | NM_016809.2 | -2.34 |
| Nupr1 | nuclear protein 1 |  | -2.35 |
| Ebaf | endometrial bleeding associated factor | NM_177099.3 | -2.40 |
| Tgfbi | transforming growth factor, beta induced | NM_009369.1 | -2.40 |
| Ppnr | per-pentamer repeat gene | NM_012022.1 | -2.44 |
| Idb1 | inhibitor of DNA binding 1 | NM_010495.1 | -2.45 |
| Tcf7l2 | transcription factor 7-like 2, T-cell specific, HMG-box | AY072035.1 | -2.47 |
| Mafb | v-maf musculoaponeurotic fibrosarcoma oncogene family, protein B (avian) | NM_010658.2 | -2.49 |
| Dhrs6 | dehydrogenase/reductase (SDR family) member 6 | NM_027208.1 | -2.60 |
| Olfml3 | olfactomedin-like 3 | NM_133859.1 | -2.61 |
| Wbscr14 | Williams-Beuren syndrome chromosome region 14 homolog (human) | NM_021455.2 | -2.69 |
| Ltbp3 | latent transforming growth factor beta binding protein 3 | NM_008520.1 | -2.79 |
| Hps6 | Hermansky-Pudlak syndrome 6 | NM_176785.1 | -4.86 |
| Fxyd5 | FXYD domain-containing ion transport regulator 5 | BC031112.1 | -18.27 |

Ac, indicates the gene accession number. Significantly regulated are those transcripts induced or repressed ≥ 2-folds vs. controls.
