# Supplementary material for: Hypoxic transcription gene profiles under the modulation of nitric oxide in nuclear run on-microarray and proteomics
Source: BMC Genomics. 2009 Sep 2;10:408. doi: 10.1186/1471-2164-10-408 (PMC2743718; doi:10.1186/1471-2164-10-408)
Supplement: Additional file 3 — Transcripts regulated after exposing cells to a combination of hypoxia and NO. Ac, indicates the gene accession number. Significantly regulated are those transcripts induced or repressed ≥ 2-folds vs. controls following the exposure to hypoxia (1% O2) plus 0.5 mM DETA-NO for 6 h. [file 1471-2164-10-408-S3.doc]

**Suppl. Table 3 - Transcripts regulated after exposing cells to a combination of hypoxia and NO**

| **UP** | **Gene name** | **Ac** | **folds** |
| --- | --- | --- | --- |
| Ddit4 | DNA-damage-inducible transcript 4 | NM_029083.1 | 35.21 |
| Trib3 | tribbles homolog 3 (Drosophila) | NM_144554.1 | 27.11 |
| Bnip3 | BCL2/adenovirus E1B 19kDa-interacting protein 1, NIP3 | NM_009760.2 | 20.06 |
| Phlda3 | pleckstrin homology-like domain, family A, member 3 | NM_013750.1 | 9.41 |
| Nqo1 | NAD(P)H dehydrogenase, quinone 1 | U12961.1 | 8.32 |
| Serpine1 | serine (or cysteine) proteinase inhibitor, clade E, member 1 | NM_008871.1 | 7.39 |
| Ndrg1 : Ndrl | N-myc downstream regulated gene 1 : N-myc downstream regulated-like | NM_010884.1 | 7.32 |
| Atf3 | activating transcription factor 3 | NM_007498.2 | 7.26 |
| Selenbp2 : Selenbp1 | selenium binding protein 2 : selenium binding protein 1 | NM_009150.2 | 6.97 |
| Vegfa | vascular endothelial growth factor A | NM_009505.2 | 6.55 |
| Hmox1 | heme oxygenase (decycling) 1 | NM_010442.1 | 6.04 |
| Sesn2 | sestrin 2 | NM_144907.1 | 5.23 |
| Adh7 | alcohol dehydrogenase 7 (class IV), mu or sigma polypeptide | NM_009626.2 | 5.06 |
| Gpt2 | glutamic pyruvate transaminase (alanine aminotransferase) 2 | NM_173866.1 | 5.02 |
| Slc7a11 | solute carrier family 7 (cationic amino acid transporter, y+ system), member 11 | NM_011990.1 | 4.74 |
| Tob1 | transducer of ErbB-2.1 | NM_009427.1 | 4.50 |
| P4ha1 | procollagen-proline, 2-oxoglutarate 4-dioxygenase (proline 4-hydroxylase), alpha 1 polypeptide |  | 4.49 |
| Fosl2 | fos-like antigen 2 | NM_008037.3 | 4.47 |
| Pgm2 | phosphoglucomutase 2 | NM_028132.2 | 4.46 |
| Hrmt1l1 | heterogeneous nuclear ribonucleoprotein methyltransferase-like 1 (S. cerevisiae) | NM_133182.1 | 4.34 |
| Cdkn1a | cyclin-dependent kinase inhibitor 1A (P21) | NM_007669.2 | 4.25 |
| Nfil3 | nuclear factor, interleukin 3, regulated | NM_017373.2 | 4.15 |
| Bnip3l | BCL2/adenovirus E1B 19kDa-interacting protein 3-like | NM_009761.2 | 3.98 |
| Hyal1 | hyaluronidase 1 | NM_008317.2 | 3.84 |
| Pcx | pyruvate carboxylase | NM_008797.1 | 3.79 |
| Pfkl | phosphofructokinase, liver, B-type | AK036318.1 | 3.69 |
| Stc2 | stanniocalcin 2 | AK002527.1 | 3.65 |
| Stc1 | stanniocalcin 1 | NM_009285.2 | 3.58 |
| Cox6a2 | cytochrome c oxidase, subunit VI a, polypeptide 2 | BC028514.1 | 3.57 |
| Scd1 | stearoyl-Coenzyme A desaturase 1 | NM_009127.2 | 3.55 |
| Cth | cystathionase (cystathionine gamma-lyase) | NM_145953.2 | 3.47 |
| Sfrs1 | splicing factor, arginine/serine-rich 1 (ASF/SF2) | NM_173374.2 | 3.42 |
| Rnf128 | ring finger protein 128 | NM_023270.3 | 3.41 |
| Gadd45a | growth arrest and DNA-damage-inducible 45 alpha | NM_007836.1 | 3.34 |
| Ier3 | immediate early response 3 | NM_133662.1 | 3.29 |
| F10 | coagulation factor X | NM_007972.2 | 3.28 |
| Gys3 : Gys1 | glycogen synthase 3, brain : glycogen synthase 1, muscle | NM_030678.2 | 3.27 |
| Grhpr | glyoxylate reductase/hydroxypyruvate reductase |  | 3.21 |
| Prss16 | protease, serine, 16 (thymus) | NM_019429.1 | 3.17 |
| Adrb2 | adrenergic receptor, beta 2 | X15643.1_CDS_1 | 3.13 |
| Myl7 | myosin, light polypeptide 7, regulatory | NM_022879.1 | 3.13 |
| Idh1 | isocitrate dehydrogenase 1 (NADP+), soluble | NM_010497.1 | 3.09 |
| Slc19a2 | solute carrier family 19 (thiamine transporter), member 2 | NM_054087.1 | 3.08 |
| Acvrl1 | activin A receptor, type II-like 1 | NM_009612.1 | 3.04 |
| Flot1 | flotillin 1 | NM_008027.1 | 3.01 |
| Neil1 | nei endonuclease VIII-like 1 (E. coli) | NM_028347.1 | 3.01 |
| Rcor1 | RE1-silencing transcription factor (REST) co-repressor 1 | NM_054048.1 | 3.00 |
| Hsd3b7 | hydroxy-delta-5-steroid dehydrogenase, 3 beta- and steroid delta-isomerase 7 | NM_133943.1 | 2.92 |
| Gdi1 | guanosine diphosphate (GDP) dissociation inhibitor 1 | NM_010273.1 | 2.92 |
| Gadd45b | growth arrest and DNA-damage-inducible 45 beta | NM_008655.1 | 2.91 |
| Dnajb4 | DnaJ (Hsp40) homolog, subfamily B, member 4 | NM_027287.1 | 2.89 |
| Ero1l | ERO1-like (S. cerevisiae) | NM_015774.2 | 2.87 |
| Tmem25 | transmembrane protein 25 | NM_027865.1 | 2.86 |
| Soat2 | sterol O-acyltransferase 2 | NM_146064.1 | 2.84 |
| Mod1 | malic enzyme, supernatant | NM_008615.1 | 2.82 |
| Efna1 | ephrin A1 | NM_010107.2 | 2.80 |
| Als2cr2 | amyotrophic lateral sclerosis 2 (juvenile) chromosome region, candidate 2 homolog (human) | NM_172656.3 | 2.76 |
| Bhlhb2 | basic helix-loop-helix domain containing, class B2 | NM_011498.2 | 2.74 |
| Sast | syntrophin associated serine/threonine kinase | NM_019945.1 | 2.72 |
| Fcgrt | Fc receptor, IgG, alpha chain transporter | NM_010189.1 | 2.71 |
| Ttll1 | tubulin tyrosine ligase-like 1 | NM_178869.2 | 2.70 |
| Pira3 | paired-Ig-like receptor A3 | NM_011090.1 | 2.68 |
| Mst1 | macrophage stimulating 1 (hepatocyte growth factor-like) | NM_008243.2 | 2.68 |
| Jmjd2b | jumonji domain containing 2B | NM_172132.1 | 2.64 |
| Asns | asparagine synthetase | NM_012055.1 | 2.63 |
| Atp6ap2 | ATPase, H+ transporting, lysosomal accessory protein 2 | NM_027439.2 | 2.62 |
| Bbc3 | Bcl-2 binding component 3 | NM_133234.1 | 2.61 |
| Txndc1 | thioredoxin domain containing 1 | NM_028339.1 | 2.58 |
| Snn | stannin | NM_009223.1 | 2.58 |
| Tiparp | TCDD-inducible poly(ADP-ribose) polymerase | NM_178892.3 | 2.56 |
| Dsip1 | delta sleep inducing peptide, immunoreactor | NM_010286.2 | 2.56 |
| Tuba1 | tubulin, alpha 1 | NM_011653.1 | 2.56 |
| Greb1 | gene regulated by estrogen in breast cancer protein | NM_015764.1 | 2.56 |
| Adam10 | a disintegrin and metalloprotease domain 10 | NM_007399.1 | 2.55 |
| Klf2 | Kruppel-like factor 2 (lung) | NM_008452.1 | 2.55 |
| Epb7.2 | erythrocyte protein band 7.2 | NM_013515.1 | 2.55 |
| Ddx5 | DEAD (Asp-Glu-Ala-Asp) box polypeptide 5 | NM_007840.1 | 2.54 |
| Tnfsf9 | tumor necrosis factor (ligand) superfamily, member 9 | NM_009404.1 | 2.53 |
| Cars | cysteinyl-tRNA synthetase | NM_013742.2 | 2.52 |
| Mx2 | myxovirus (influenza virus) resistance 2 | NM_013606.1 | 2.52 |
| Sertad1 | SERTA domain containing 1 | NM_018820.3 | 2.51 |
| Ddit3 | DNA-damage inducible transcript 3 | NM_007837.2 | 2.51 |
| H2-T9 : H2-T22 : H2-T17 : H2-T10 | histocompatibility 2, T region locus 9 : histocompatibility 2, T region locus 22 : histocompatibility 2, T region locus 17 : histocompatibility 2, T region locus 10 | NM_010399.2 | 2.51 |
| Mdm2 | transformed mouse 3T3 cell double minute 2 | X58876.1 | 2.50 |
| Zfx : Zfa | zinc finger protein X-linked : zinc finger protein, autosomal | NM_011768.1 | 2.49 |
| Mthfd2 | methylenetetrahydrofolate dehydrogenase (NAD+ dependent), methenyltetrahydrofolate cyclohydrolase | NM_008638.1 | 2.49 |
| Lss | lanosterol synthase | AK012813.1 | 2.48 |
| Btg2 | B-cell translocation gene 2, anti-proliferative | NM_007570.1 | 2.48 |
| Amhr2 | anti-Mullerian hormone type 2 receptor | NM_144547.1 | 2.47 |
| Dgat2 | diacylglycerol O-acyltransferase 2 | NM_026384.2 | 2.47 |
| Bcl6 | B-cell leukemia/lymphoma 6 | NM_009744.2 | 2.47 |
| Egln1 | EGL nine homolog 1 (C. elegans) | NM_053207.1 | 2.46 |
| Pmaip1 | phorbol-12-myristate-13-acetate-induced protein 1 | NM_021451.1 | 2.46 |
| Npn3 | neoplastic progression 3 | NM_029688.2 | 2.46 |
| Mod1 | malic enzyme, supernatant | NM_008615.1 | 2.45 |
| Eif4g2 | eukaryotic translation initiation factor 4, gamma 2 | NM_013507.2 | 2.45 |
| Slc37a4 | solute carrier family 37 (glycerol-6-phosphate transporter), member 4 | NM_008063.1 | 2.44 |
| Ceacam1 | CEA-related cell adhesion molecule 1 | NM_011926.1 | 2.43 |
| Abcd3 | ATP-binding cassette, sub-family D (ALD), member 3 | NM_008991.1 | 2.43 |
| Pira3 | paired-Ig-like receptor A3 | NM_011090.1 | 2.42 |
| Sgpp1 | sphingosine-1-phosphate phosphatase 1 | NM_030750.2 | 2.42 |
| Tmem2 | transmembrane protein 2 | NM_031997.2 | 2.42 |
| Prkar2b | protein kinase, cAMP dependent regulatory, type II beta | NM_011158.2 | 2.42 |
| Pgk1 | phosphoglycerate kinase 1 | NM_008828.1 | 2.41 |
| Dusp1 | dual specificity phosphatase 1 | NM_013642.1 | 2.41 |
| Pbef1 | pre-B-cell colony-enhancing factor 1 | NM_021524.1 | 2.40 |
| Slc16a3 | solute carrier family 16 (monocarboxylic acid transporters), member 3 | NM_030696.2 | 2.40 |
| Mxd4 | Max dimerization protein 4 | NM_010753.2 | 2.39 |
| Atrn | attractin | NM_009730.1 | 2.39 |
| Impact | imprinted and ancient | NM_008378.1 | 2.39 |
| Smpd1 | sphingomyelin phosphodiesterase 1, acid lysosomal | NM_011421.1 | 2.38 |
| Aldh6a1 | aldehyde dehydrogenase family 6, subfamily A1 | NM_134042.1 | 2.37 |
| Osp94 | osmotic stress protein | NM_011020.3 | 2.37 |
| Myd116 | myeloid differentiation primary response gene 116 | NM_008654.1 | 2.37 |
| Slc2a8 | solute carrier family 2, (facilitated glucose transporter), member 8 | NM_019488.2 | 2.37 |
| Zfp367 | zinc finger protein 367 | NM_175494.2 | 2.35 |
| Nxf7 | nuclear RNA export factor 7 | NM_130888.1 | 2.35 |
| Nars | asparaginyl-tRNA synthetase | NM_027350.1 | 2.35 |
| Cmya1 | cardiomyopathy associated 1 | NM_011724.1 | 2.34 |
| Cat | catalase | NM_009804.1 | 2.34 |
| Gp49b | glycoprotein 49 B | NM_013532.1 | 2.34 |
| Pgk1 | phosphoglycerate kinase 1 | NM_008828.1 | 2.34 |
| Rbl2 | retinoblastoma-like 2 | NM_011250.2 | 2.33 |
| Gm2a | GM2 ganglioside activator protein | NM_010299.2 | 2.33 |
| Tuft1 | tuftelin 1 | NM_011656.1 | 2.32 |
| Ceacam1 | CEA-related cell adhesion molecule 1 | NM_011926.1 | 2.32 |
| Fdps | farnesyl diphosphate synthetase | AK077979.1 | 2.32 |
| Il15 | interleukin 15 | NM_008357.1 | 2.31 |
| Angptl6 | angiopoietin-like 6 | NM_145154.1 | 2.30 |
| Cbs | cystathionine beta-synthase | NM_144855.1 | 2.30 |
| D4Ertd765e | DNA segment, Chr 4, ERATO Doi 765, expressed | NM_026728.1 | 2.30 |
| Tenc1 | tensin like C1 domain-containing phosphatase | NM_153533.1 | 2.28 |
| Serpinb12 | serine (or cysteine) proteinase inhibitor, clade B (ovalbumin), member 12 | NM_027971.1 | 2.27 |
| Bnip3l | BCL2/adenovirus E1B 19kDa-interacting protein 3-like | NM_009761.2 | 2.26 |
| Mgl1 | macrophage galactose N-acetyl-galactosamine specific lectin 1 | NM_010796.1 | 2.26 |
| Mmp11 | matrix metalloproteinase 11 | NM_008606.1 | 2.26 |
| Chpt1 | choline phosphotransferase 1 | NM_144807.2 | 2.25 |
| Pgam1 | phosphoglycerate mutase 1 | NM_023418.1 | 2.25 |
| Sparc | secreted acidic cysteine rich glycoprotein | NM_009242.1 | 2.25 |
| Vamp2 | vesicle-associated membrane protein 2 | NM_009497.2 | 2.25 |
| Gpnmb | glycoprotein (transmembrane) nmb | NM_053110.2 | 2.23 |
| Sqrdl | sulfide quinone reductase-like (yeast) | NM_021507.4 | 2.23 |
| Vcl | vinculin | NM_009502.3 | 2.22 |
| Pfkm | phosphofructokinase, muscle | NM_021514.2 | 2.22 |
| Pdcd4 | programmed cell death 4 | NM_011050.1 | 2.22 |
| Picalm | phosphatidylinositol binding clathrin assembly protein | NM_146194.2 | 2.22 |
| Bat4 | HLA-B associated transcript 4 | NM_032460.1 | 2.22 |
| Aplp2 | amyloid beta (A4) precursor-like protein 2 | M97216.1 | 2.21 |
| Zfp101 | zinc finger protein 101 | NM_009542.1 | 2.21 |
| Zfp292 | zinc finger protein 292 | NM_013889.1 | 2.21 |
| Traf4 | Tnf receptor associated factor 4 | NM_009423.2 | 2.20 |
| Zfp336 | zinc finger protein 336 | NM_028986.1 | 2.20 |
| Cyp2c55 | cytochrome P450, family 2, subfamily c, polypeptide 55 | NM_028089.1 | 2.20 |
| Col4a3bp | procollagen, type IV, alpha 3 (Goodpasture antigen) binding protein | NM_023420.1 | 2.20 |
| Plod1 | procollagen-lysine, 2-oxoglutarate 5-dioxygenase 1 | NM_011122.1 | 2.20 |
| Tpi | triosephosphate isomerase | NM_009415.1 | 2.20 |
| Hebp1 | heme binding protein 1 | AF117613.1 | 2.19 |
| Aldo1 | aldolase 1, A isoform | NM_007438.2 | 2.19 |
| Pabpc1 | poly A binding protein, cytoplasmic 1 | NM_008774.2 | 2.19 |
| Slc25a30 | solute carrier family 25, member 30 | AK090086.1 | 2.19 |
| Gdap10 | ganglioside-induced differentiation-associated-protein 10 | NM_010268.1 | 2.19 |
| Ass1 | argininosuccinate synthetase 1 | NM_007494.2 | 2.17 |
| 4921511C16 | hypothetical protein 4921511C16 | NM_183307.1 | 2.17 |
| Tec | cytoplasmic tyrosine kinase, Dscr28C related (Drosophila) | NM_013689.2 | 2.17 |
| Rnf13 | ring finger protein 13 | NM_011883.1 | 2.17 |
| Zfp68 | zinc finger protein 68 | NM_013844.1 | 2.16 |
| Smox | spermine oxidase | NM_145533.1 | 2.16 |
| Alas1 | aminolevulinic acid synthase 1 | NM_020559.1 | 2.16 |
| Aldo1 | aldolase 1, A isoform | NM_007438.2 | 2.15 |
| Golph3 | golgi phosphoprotein 3 | NM_025673.2 | 2.15 |
| Gcl | germ cell-less homolog (Drosophila) | NM_011818.2 | 2.14 |
| Twsg1 | twisted gastrulation homolog 1 (Drosophila) | NM_023053.1 | 2.14 |
| Nical | NEDD9 interacting protein with calponin homology and LIM domains | NM_138315.1 | 2.14 |
| Sep-06 | septin 6 | NM_019942.2 | 2.14 |
| Pdxp | pyridoxal (pyridoxine, vitamin B6) phosphatase | NM_020271.2 | 2.14 |
| Scp2 | sterol carrier protein 2, liver | NM_011327.1 | 2.14 |
| Adfp | adipose differentiation related protein | NM_007408.2 | 2.14 |
| Tmc4 | transmembrane channel-like gene family 4 | NM_181820.1 | 2.14 |
| Zfp108 | zinc finger protein 108 | NM_018791.1 | 2.13 |
| Tnfaip2 | tumor necrosis factor, alpha-induced protein 2 | NM_009396.1 | 2.13 |
| Rbbp9 | retinoblastoma binding protein 9 | NM_015754.1 | 2.13 |
| Sdcbp | syndecan binding protein | NM_016807.1 | 2.13 |
| Abcb1b | ATP-binding cassette, sub-family B (MDR/TAP), member 1B | NM_011075.1 | 2.13 |
| Akr1b3 | aldo-keto reductase family 1, member B3 (aldose reductase) | NM_009658.2 | 2.13 |
| Slc11a1 | solute carrier family 11 (proton-coupled divalent metal ion transporters), member 1 | NM_013612.1 | 2.13 |
| Olfr1395 | olfactory receptor 1395 | NM_146877.1 | 2.12 |
| Dip3b | Dip3 beta | NM_145220.1 | 2.11 |
| Lrrc1 | leucine rich repeat containing 1 | NM_172528.2 | 2.11 |
| Rrm1 | ribonucleotide reductase M1 | NM_009103.2 | 2.11 |
| Pigh | phosphatidylinositol glycan, class H | NM_029988.1 | 2.11 |
| Pias3 | protein inhibitor of activated STAT 3 | NM_018812.1 | 2.10 |
| Acat3 | acetyl-Coenzyme A acetyltransferase 3 | NM_153151.1 | 2.09 |
| Cbx1 | chromobox homolog 1 (Drosophila HP1 beta) |  | 2.09 |
| Slc30a9 | solute carrier family 30 (zinc transporter), member 9 | NM_178651.2 | 2.09 |
| Hspd1 | heat shock protein 1 (chaperonin) | NM_010477.2 | 2.09 |
| Usp18 | ubiquitin specific protease 18 | NM_011909.1 | 2.09 |
| Gpsm1 | G-protein signalling modulator 1 (AGS3-like, C. elegans) | NM_153410.2 | 2.08 |
| Pqlc2 | PQ loop repeat containing 2 | NM_145384.1 | 2.08 |
| Pycr1 | pyrroline-5-carboxylate reductase 1 | NM_144795.1 | 2.08 |
| Esd | esterase D/formylglutathione hydrolase | NM_016903.2 | 2.08 |
| Uxs1 | UDP-glucuronate decarboxylase 1 | NM_026430.1 | 2.07 |
| Slc27a4 | solute carrier family 27 (fatty acid transporter), member 4 | NM_011989.1 | 2.07 |
| Galnact2 | chondroitin sulfate GalNAcT-2 | NM_030165.2 | 2.07 |
| Fcgrt | Fc receptor, IgG, alpha chain transporter | NM_010189.1 | 2.06 |
| Abcd4 | ATP-binding cassette, sub-family D (ALD), member 4 | NM_008992.1 | 2.05 |
| Tpmt | thiopurine methyltransferase | NM_016785.1 | 2.05 |
| Irf7 | interferon regulatory factor 7 | NM_016850.1 | 2.05 |
| Oas1b | 2'-5' oligoadenylate synthetase 1B | NM_011853.1 | 2.05 |
| Nsmaf | neutral sphingomyelinase (N-SMase) activation associated factor | NM_010945.1 | 2.05 |
| Rad23b | RAD23b homolog (S. cerevisiae) | NM_009011.2 | 2.04 |
| Zfp146 | zinc finger protein 146 | NM_011980.1 | 2.04 |
| Nxn | nucleoredoxin | NM_008750.2 | 2.04 |
| Fcgr2b | Fc receptor, IgG, low affinity IIb | NM_010187.1 | 2.04 |
| Clk | CDC-like kinase | NM_009905.1 | 2.04 |
| Nfe2l1 | nuclear factor, erythroid derived 2,-like 1 | NM_008686.2 | 2.03 |
| Dirc2 | disrupted in renal carcinoma 2 (human) | NM_153550.2 | 2.03 |
| Nipsnap3b | nipsnap homolog 3B (C. elegans) | NM_025623.1 | 2.03 |
| Ncoa4 | nuclear receptor coactivator 4 | NM_019744.1 | 2.02 |
| Mpv17l | Mpv17 transgene, kidney disease mutant-like | NM_033564.1 | 2.02 |
| Btg1 | B-cell translocation gene 1, anti-proliferative | NM_007569.1 | 2.02 |
| Setmar | SET domain and mariner transposase fusion gene | NM_178391.2 | 2.02 |
| Sla | src-like adaptor | NM_009192.1 | 2.01 |
| Abhd4 | abhydrolase domain containing 4 | NM_134076.1 | 2.01 |
| Sdh1 | sorbitol dehydrogenase 1 | NM_146126.1 | 2.01 |

| **DOWN** | **Gene name** | **Ac** | **Folds** |
| --- | --- | --- | --- |
| Tnfrsf11a | tumor necrosis factor receptor superfamily, member 11a | NM_009399.2 | -2.01 |
| Zcchc11 | zinc finger, CCHC domain containing 11 | NM_175472.2 | -2.02 |
| Ssbp1 | single-stranded DNA binding protein 1 | NM_212468.3 | -2.02 |
| Tal1 | T-cell acute lymphocytic leukemia 1 | NM_011527.1 | -2.02 |
| Ctdspl | CTD (carboxy-terminal domain, RNA polymerase II, polypeptide A) small phosphatase-like | NM_133710.1 | -2.03 |
| Mrpl52 | mitochondrial ribosomal protein L52 | NM_026851.1 | -2.06 |
| Armet | arginine-rich, mutated in early stage tumors | NM_029103.1 | -2.07 |
| Pabpc1 | poly A binding protein, cytoplasmic 1 | NM_008774.2 | -2.07 |
| Mbtps1 | membrane-bound transcription factor protease, site 1 | AK002809.1 | -2.07 |
| Crip1 | cysteine-rich protein 1 (intestinal) | NM_007763.1 | -2.09 |
| Hist1h1b | histone 1, H1b | NM_020034.1 | -2.12 |
| Trerf1 | transcriptional regulating factor 1 | NM_172622.1 | -2.12 |
| Arid3a | AT rich interactive domain 3A (Bright like) | NM_007880.1 | -2.13 |
| Lfng | lunatic fringe gene homolog (Drosophila) | AK004642.1 | -2.13 |
| Prtn3 | proteinase 3 | NM_011178.2 | -2.14 |
| Rps15a | ribosomal protein S15a | NM_170669.2 | -2.14 |
| Plk1 | polo-like kinase 1 (Drosophila) | NM_011121.2 | -2.15 |
| U2af1-rs2 | U2 small nuclear ribonucleoprotein auxiliary factor (U2AF) 1, related sequence 2 | NM_178794.2 | -2.17 |
| Rps19 | ribosomal protein S19 | NM_023133.1 | -2.18 |
| Pes1 | pescadillo homolog 1, containing BRCT domain (zebrafish) | NM_022889.2 | -2.19 |
| Son | Son cell proliferation protein | NM_178880.3 | -2.23 |
| Fau | Finkel-Biskis-Reilly murine sarcoma virus (FBR-MuSV) ubiquitously expressed (fox derived) | NM_007990.1 | -2.24 |
| 6530401P13 | hypothetical protein 6530401P13 | NM_177644.2 | -2.25 |
| Cks2 | CDC28 protein kinase regulatory subunit 2 | NM_025415.1 | -2.25 |
| Wasf2 | WAS protein family, member 2 | NM_153423.3 | -2.26 |
| Nvl | nuclear VCP-like | NM_026171.1 | -2.27 |
| Wdr5b | WD repeat domain 5B | NM_027113.2 | -2.28 |
| Mrpl34 | mitochondrial ribosomal protein L34 | NM_053162.1 | -2.31 |
| Fkbp11 | FK506 binding protein 11 | NM_024169.2 | -2.31 |
| Gpr35 | G protein-coupled receptor 35 | NM_022320.2 | -2.34 |
| Bcl2a1c | B-cell leukemia/lymphoma 2 related protein A1c | NM_007535.1 | -2.34 |
| Ank2 | ankyrin 2, brain | NM_178655.2 | -2.35 |
| Idb1 | inhibitor of DNA binding 1 | NM_010495.1 | -2.36 |
| Scgf | stem cell growth factor | NM_009131.1 | -2.37 |
| Bcl2a1d : Bcl2a1b : Bcl2a1a | B-cell leukemia/lymphoma 2 related protein A1d : B-cell leukemia/lymphoma 2 related protein A1b : B-cell leukemia/lymphoma 2 related protein A1a | NM_007536.1 | -2.40 |
| Timm13a | translocase of inner mitochondrial membrane 13 homolog a (yeast) | NM_013899.1 | -2.41 |
| Ptgs1 | prostaglandin-endoperoxide synthase 1 | NM_008969.1 | -2.41 |
| Cks2 | CDC28 protein kinase regulatory subunit 2 | NM_025415.1 | -2.41 |
| Nnt | nicotinamide nucleotide transhydrogenase | AK087064.1 | -2.41 |
| Dhrs6 | dehydrogenase/reductase (SDR family) member 6 | NM_027208.1 | -2.42 |
| Tle6 | transducin-like enhancer of split 6, homolog of Drosophila E(spl) | NM_053254.1 | -2.43 |
| Mtf1 | metal response element binding transcription factor 1 | NM_008636.2 | -2.44 |
| Egr1 | early growth response 1 | NM_007913.2 | -2.44 |
| Olr1 | oxidized low density lipoprotein (lectin-like) receptor 1 | NM_138648.1 | -2.47 |
| Pilra | paired immunoglobin-like type 2 receptor alpha | NM_153510.1 | -2.51 |
| Clecsf12 | C-type (calcium dependent, carbohydrate recognition domain) lectin, superfamily member 12 | NM_020008.1 | -2.54 |
| Lst1 | leukocyte specific transcript 1 | NM_010734.1 | -2.55 |
| Trim17 | tripartite motif protein 17 | NM_031172.1 | -2.57 |
| Ctsw | cathepsin W | NM_009985.2 | -2.58 |
| Timm9 | translocase of inner mitochondrial membrane 9 homolog (yeast) | NM_013895.2 | -2.59 |
| Gpr84 | G protein-coupled receptor 84 | NM_030720.1 | -2.67 |
| Tbxa2r | thromboxane A2 receptor | NM_009325.1 | -2.69 |
| Cxcl14 | chemokine (C-X-C motif) ligand 14 | AK004615.1 | -2.73 |
| Snrpg | small nuclear ribonucleoprotein polypeptide G | NM_026506.1 | -2.73 |
| Rps27a | ribosomal protein S27a | NM_024277.1 | -2.83 |
| Ltb | lymphotoxin B | NM_008518.1 | -2.86 |
| Hoxa7 | homeo box A7 | NM_010455.1 | -2.88 |
| Uqcr | ubiquinol-cytochrome c reductase (6.4kD) subunit | NM_025650.1 | -3.00 |
| Rpl41 | ribosomal protein L41 | NM_018860.2 | -3.00 |
| Hist1h2ak | histone 1, H2ak | NM_178183.1 | -3.22 |
| Hist2h2aa1 : Hist2h2ac : Hist2h2ab | histone 2, H2aa1 : histone 2, H2ac : histone 2, H2ab | NM_178213.2 | -3.22 |
| Hist1h2ab : Hist1h2ad | histone 1, H2ab : histone 1, H2ad | NM_178188.1 | -3.29 |
| Hip1 | huntingtin interacting protein 1 | NM_146001.1 | -3.38 |
| Hist1h2af : Hist1h2ae | histone 1, H2af : histone 1, H2ae | NM_175661.1 | -3.39 |
| Hist1h2an | histone 1, H2an | NM_178184.1 | -3.82 |
| Hist1h2ao | histone 1, H2ao | NM_178185.1 | -3.84 |
| Hps6 | Hermansky-Pudlak syndrome 6 | NM_176785.1 | -4.21 |
| Fxyd5 | FXYD domain-containing ion transport regulator 5 | BC031112.1 | -9.71 |

Ac, indicates the gene accession number. Significantly regulated are those transcripts induced or repressed ≥ 2-folds vs. controls following the exposure to hypoxia (1% O_2_) plus 0.5 mM DETA-NO for 6 h.
