# Supplementary material for: Hypoxic transcription gene profiles under the modulation of nitric oxide in nuclear run on-microarray and proteomics
Source: BMC Genomics. 2009 Sep 2;10:408. doi: 10.1186/1471-2164-10-408 (PMC2743718; doi:10.1186/1471-2164-10-408)
Supplement: Additional file 4 — Impact of NO on genes regulated by hypoxia. This list compared gene profiles generated by vs. that generated by hypoxia and NO. Hyp = hypoxic treatment (1% O2); NO = nitric oxide treatment (0.5 mM DETA-NO). [file 1471-2164-10-408-S4.doc]

**Stable 1** - **Impact of NO on genes regulated by hypoxia**

| **Symbol** | **Gene Name** | **Ac** | **Hyp** | **Hyp + NO** |
| --- | --- | --- | --- | --- |
| Bnip3 | BCL2/adenovirus E1B 19kDa-interacting protein 1, NIP3 | NM_009760.2 | 33.40 | 20.06 |
| Selenbp2 : Selenbp1 | selenium binding protein 2 : selenium binding protein 1 | NM_009150.2 | 23.96 | 6.97 |
| Ddit4 | DNA-damage-inducible transcript 4 | NM_029083.1 | 19.68 | 35.21 |
| Ndrg1 : Ndrl | N-myc downstream regulated gene 1 : N-myc downstream regulated-like | NM_010884.1 | 13.18 | 7.32 |
| Vegfa | vascular endothelial growth factor A | NM_009505.2 | 9.08 | 6.55 |
| Serpine1 | serine (or cysteine) proteinase inhibitor, clade E, member 1 | NM_008871.1 | 8.06 | 7.39 |
| Trib3 | tribbles homolog 3 (Drosophila) | NM_144554.1 | 8.05 | 27.11 |
| Atf3 | activating transcription factor 3 | NM_007498.2 | 7.27 | 7.26 |
| Ier3 | immediate early response 3 | NM_133662.1 | 7.21 | 3.29 |
| F10 | coagulation factor X | NM_007972.2 | 7.06 | 3.28 |
| Gys3 : Gys1 | glycogen synthase 3, brain : glycogen synthase 1, muscle | NM_030678.2 | 6.30 | 3.27 |
| Pgm2 | phosphoglucomutase 2 | NM_028132.2 | 6.24 | 4.46 |
| Hmox1 | heme oxygenase (decycling) 1 | NM_010442.1 | 6.14 | 6.04 |
| Rcor1 | RE1-silencing transcription factor (REST) co-repressor 1 | NM_054048.1 | 5.69 | 3.00 |
| Hyal1 | hyaluronidase 1 | NM_008317.2 | 5.09 | 3.84 |
| Egln1 | EGL nine homolog 1 (C. elegans) | NM_053207.1 | 4.99 | 2.46 |
| Pfkl | phosphofructokinase, liver, B-type | AK036318.1 | 4.99 | 3.69 |
| Grhpr | glyoxylate reductase/hydroxypyruvate reductase |  | 4.75 | 3.21 |
| P4ha1 | procollagen-proline, 2-oxoglutarate 4-dioxygenase (proline 4-hydroxylase), alpha 1 polypeptide |  | 4.52 | 4.49 |
| Ero1l | ERO1-like (S. cerevisiae) | NM_015774.2 | 4.28 | 2.87 |
| Plod1 | procollagen-lysine, 2-oxoglutarate 5-dioxygenase 1 | NM_011122.1 | 4.12 | 2.20 |
| Slc16a3 | solute carrier family 16 (monocarboxylic acid transporters), member 3 | NM_030696.2 | 4.11 | 2.40 |
| Cox6a2 | cytochrome c oxidase, subunit VI a, polypeptide 2 | BC028514.1 | 4.07 | 3.57 |
| Myl7 | myosin, light polypeptide 7, regulatory | NM_022879.1 | 3.93 | 3.13 |
| Rnf128 | ring finger protein 128 | NM_023270.3 | 3.86 | 3.41 |
| Nfil3 | nuclear factor, interleukin 3, regulated | NM_017373.2 | 3.79 | 4.15 |
| Amhr2 | anti-Mullerian hormone type 2 receptor | NM_144547.1 | 3.72 | 2.47 |
| Bnip3l | BCL2/adenovirus E1B 19kDa-interacting protein 3-like | NM_009761.2 | 3.72 | 3.98 |
| Stc2 | stanniocalcin 2 | AK002527.1 | 3.63 | 3.65 |
| Slc37a4 | solute carrier family 37 (glycerol-6-phosphate transporter), member 4 | NM_008063.1 | 3.55 | 2.44 |
| Jmjd2b | jumonji domain containing 2B | NM_172132.1 | 3.51 | 2.64 |
| Tob1 | transducer of ErbB-2.1 | NM_009427.1 | 3.50 | 4.50 |
| Bhlhb2 | basic helix-loop-helix domain containing, class B2 | NM_011498.2 | 3.47 | 2.74 |
| Bnip3l | BCL2/adenovirus E1B 19kDa-interacting protein 3-like | NM_009761.2 | 3.33 | 2.26 |
| Dip3b | Dip3 beta | NM_145220.1 | 3.27 | 2.11 |
| Mx2 | myxovirus (influenza virus) resistance 2 | NM_013606.1 | 3.17 | 2.52 |
| Vamp2 | vesicle-associated membrane protein 2 | NM_009497.2 | 3.01 | 2.25 |
| Gdap10 | ganglioside-induced differentiation-associated-protein 10 | NM_010268.1 | 2.99 | 2.19 |
| Pgk1 | phosphoglycerate kinase 1 | NM_008828.1 | 2.89 | 2.41 |
| Cdkn1a | cyclin-dependent kinase inhibitor 1A (P21) | NM_007669.2 | 2.89 | 4.25 |
| Efna1 | ephrin A1 | NM_010107.2 | 2.85 | 2.80 |
| Pgk1 | phosphoglycerate kinase 1 | NM_008828.1 | 2.81 | 2.34 |
| Stc1 | stanniocalcin 1 | NM_009285.2 | 2.80 | 3.58 |
| Irf7 | interferon regulatory factor 7 | NM_016850.1 | 2.77 | 2.05 |
| Gm2a | GM2 ganglioside activator protein | NM_010299.2 | 2.75 | 2.33 |
| Smox | spermine oxidase | NM_145533.1 | 2.69 | 2.16 |
| Pgam1 | phosphoglycerate mutase 1 | NM_023418.1 | 2.68 | 2.25 |
| Acvrl1 | activin A receptor, type II-like 1 | NM_009612.1 | 2.65 | 3.04 |
| Pbef1 | pre-B-cell colony-enhancing factor 1 | NM_021524.1 | 2.64 | 2.40 |
| Tpi | triosephosphate isomerase | NM_009415.1 | 2.62 | 2.20 |
| Scd1 | stearoyl-Coenzyme A desaturase 1 | NM_009127.2 | 2.54 | 3.55 |
| D4Ertd765e | DNA segment, Chr 4, ERATO Doi 765, expressed | NM_026728.1 | 2.52 | 2.30 |
| Sertad1 | SERTA domain containing 1 | NM_018820.3 | 2.51 | 2.51 |
| Tiparp | TCDD-inducible poly(ADP-ribose) polymerase | NM_178892.3 | 2.48 | 2.56 |
| Pdxp | pyridoxal (pyridoxine, vitamin B6) phosphatase | NM_020271.2 | 2.46 | 2.14 |
| Fosl2 | fos-like antigen 2 | NM_008037.3 | 2.42 | 4.47 |
| Il15 | interleukin 15 | NM_008357.1 | 2.41 | 2.31 |
| Prss16 | protease, serine, 16 (thymus) | NM_019429.1 | 2.40 | 3.17 |
| Zfp292 | zinc finger protein 292 | NM_013889.1 | 2.40 | 2.21 |
| Ncoa4 | nuclear receptor coactivator 4 | NM_019744.1 | 2.37 | 2.02 |
| Nxn | nucleoredoxin | NM_008750.2 | 2.35 | 2.04 |
| Sesn2 | sestrin 2 | NM_144907.1 | 2.32 | 5.23 |
| Oas1b | 2'-5' oligoadenylate synthetase 1B | NM_011853.1 | 2.31 | 2.05 |
| Cbs | cystathionine beta-synthase | NM_144855.1 | 2.28 | 2.30 |
| Pira3 | paired-Ig-like receptor A3 | NM_011090.1 | 2.23 | 2.68 |
| Aldo1 | aldolase 1, A isoform | NM_007438.2 | 2.22 | 2.15 |
| Aldo1 | aldolase 1, A isoform | NM_007438.2 | 2.20 | 2.19 |
| Dgat2 | diacylglycerol O-acyltransferase 2 | NM_026384.2 | 2.18 | 2.47 |
| Hsd3b7 | hydroxy-delta-5-steroid dehydrogenase, 3 beta- and steroid delta-isomerase 7 | NM_133943.1 | 2.18 | 2.92 |
| Tmem25 | transmembrane protein 25 | NM_027865.1 | 2.17 | 2.86 |
| Adfp | adipose differentiation related protein | NM_007408.2 | 2.16 | 2.14 |
| Nxf7 | nuclear RNA export factor 7 | NM_130888.1 | 2.15 | 2.35 |
| Pias3 | protein inhibitor of activated STAT 3 | NM_018812.1 | 2.14 | 2.10 |
| Usp18 | ubiquitin specific protease 18 | NM_011909.1 | 2.13 | 2.09 |
| Galnact2 | chondroitin sulfate GalNAcT-2 | NM_030165.2 | 2.10 | 2.07 |
| Pira3 | paired-Ig-like receptor A3 | NM_011090.1 | 2.07 | 2.42 |
| Atrn | attractin | NM_009730.1 | 2.03 | 2.39 |
| Als2cr2 | amyotrophic lateral sclerosis 2 (juvenile) chromosome region, candidate 2 homolog (human) | NM_172656.3 | 2.02 | 2.76 |
| Btg2 | B-cell translocation gene 2, anti-proliferative | NM_007570.1 | 2.01 | 2.48 |
| Fcgrt | Fc receptor, IgG, alpha chain transporter | NM_010189.1 | 2.00 | 2.71 |
| Hist1h1b | histone 1, H1b | NM_020034.1 | -2.10 | -2.12 |
| Armet | arginine-rich, mutated in early stage tumors | NM_029103.1 | -2.12 | -2.07 |
| Son | Son cell proliferation protein | NM_007990.1 | -2.16 | -2.23 |
| Wdr5b | WD repeat domain 5B | NM_027113.2 | -2.18 | -2.28 |
| Mrpl52 | mitochondrial ribosomal protein L52 | NM_026851.1 | -2.21 | -2.06 |
| Nnt | nicotinamide nucleotide transhydrogenase | AK087064.1 | -2.39 | -2.41 |
| Trim17 | tripartite motif protein 17 | NM_031172.1 | -2.44 | -2.57 |
| Hist1h2ab : Hist1h2ad | histone 1, H2ab : histone 1, H2ad | NM_178188.1 | -2.47 | -3.29 |
| Clecsf12 | C-type (calcium dependent, carbohydrate recognition domain) lectin, superfamily member 12 | NM_020008.1 | -2.61 | -2.54 |
| Lst1 | leukocyte specific transcript 1 | NM_010734.1 | -2.62 | -2.55 |
| Hist1h2ao | histone 1, H2ao | NM_178185.1 | -2.67 | -3.84 |
| Hist1h2af : Hist1h2ae | histone 1, H2af : histone 1, H2ae | NM_175661.1 | -2.67 | 3.39 |
| Hist1h2ak | histone 1, H2ak | NM_178183.1 | -2.68 | -3.22 |
| Hist1h2an | histone 1, H2an | NM_178184.1 | -2.71 | -3.82 |
| Hist2h2aa1 : Hist2h2ac : Hist2h2ab | histone 2, H2aa1 : histone 2, H2ac : histone 2, H2ab | NM_178213.2 | -2.73 | -3.22 |
| Bcl2a1c | B-cell leukemia/lymphoma 2 related protein A1c | NM_007535.1 | -2.91 | -2.34 |
| Bcl2a1d : Bcl2a1b : Bcl2a1a | B-cell leukemia/lymphoma 2 related protein A1d : B-cell leukemia/lymphoma 2 related protein A1b : B-cell leukemia/lymphoma 2 related protein A1a | NM_007536.1 | -3.22 | -2.40 |
| Egr1 | early growth response 1 | NM_007913.2 | -3.27 | -2.44 |
| Hps6 | Hermansky-Pudlak syndrome 6 | NM_176785.1 | -3.74 | -4.21 |
| Fxyd5 | FXYD domain-containing ion transport regulator 5 | BC031112.1 | -4.20 | -9.71 |

This list compared gene profiles generated by vs. that generated by hypoxia and NO.

Hyp = hypoxic treatment (1% O_2_); NO = nitric oxide treatment (0.5 mM DETA-NO).
