# Supplementary material for: Hypoxic transcription gene profiles under the modulation of nitric oxide in nuclear run on-microarray and proteomics
Source: BMC Genomics. 2009 Sep 2;10:408. doi: 10.1186/1471-2164-10-408 (PMC2743718; doi:10.1186/1471-2164-10-408)
Supplement: Additional file 5 — Transcripts unique to the treatment of cells with the combination of hypoxia and NO. The cross-talk between hypoxia and NO generated 162 transcripts unique to treatment of cells with the combination of hypoxia and NO. Hyp = hypoxic treatment (1% O2); NO = nitric oxide treatment (0.5 mM DETA-NO). [file 1471-2164-10-408-S5.doc]

**Stable 2** – **Transcripts unique to the treatment of cells with the combination of hypoxia and NO**

| **Symbol** | **Gene Name** | **Ac** | **Hyp + NO** |
| --- | --- | --- | --- |
| Pcx | pyruvate carboxylase | NM_008797.1 | 3.79 |
| Cth | cystathionase (cystathionine gamma-lyase) | NM_145953.2 | 3.47 |
| Gadd45a | growth arrest and DNA-damage-inducible 45 alpha | NM_007836.1 | 3.34 |
| Adrb2 | adrenergic receptor, beta 2 | X15643.1_CDS_1 | 3.13 |
| Idh1 | isocitrate dehydrogenase 1 (NADP+), soluble | NM_010497.1 | 3.09 |
| Flot1 | flotillin 1 | NM_008027.1 | 3.01 |
| Neil1 | nei endonuclease VIII-like 1 (E. coli) | NM_028347.1 | 3.01 |
| Gdi1 | guanosine diphosphate (GDP) dissociation inhibitor 1 | NM_010273.1 | 2.92 |
| Gadd45b | growth arrest and DNA-damage-inducible 45 beta | NM_008655.1 | 2.91 |
| Dnajb4 | DnaJ (Hsp40) homolog, subfamily B, member 4 | NM_025926.1 | 2.89 |
| Soat2 | sterol O-acyltransferase 2 | NM_146064.1 | 2.84 |
| Mod1 | malic enzyme, supernatant | NM_008615.1 | 2.82 |
| Sast | syntrophin associated serine/threonine kinase | NM_019945.1 | 2.72 |
| Ttll1 | tubulin tyrosine ligase-like 1 | NM_178869.2 | 2.70 |
| Mst1 | macrophage stimulating 1 (hepatocyte growth factor-like) | NM_008243.2 | 2.68 |
| Asns | asparagine synthetase | NM_012055.1 | 2.63 |
| Bbc3 | Bcl-2 binding component 3 | NM_133234.1 | 2.61 |
| Snn | stannin | NM_009223.1 | 2.58 |
| Dsip1 | delta sleep inducing peptide, immunoreactor | NM_010286.2 | 2.56 |
| Tuba1 | tubulin, alpha 1 | NM_011653.1 | 2.56 |
| Greb1 | gene regulated by estrogen in breast cancer protein | NM_015764.1 | 2.56 |
| Klf2 | Kruppel-like factor 2 (lung) | NM_008452.1 | 2.55 |
| Epb7.2 | erythrocyte protein band 7.2 | NM_013515.1 | 2.55 |
| Ddx5 | DEAD (Asp-Glu-Ala-Asp) box polypeptide 5 | NM_007840.1 | 2.54 |
| Tnfsf9 | tumor necrosis factor (ligand) superfamily, member 9 | NM_009404.1 | 2.53 |
| Cars | cysteinyl-tRNA synthetase | NM_013742.2 | 2.52 |
| Ddit3 | DNA-damage inducible transcript 3 | NM_007837.2 | 2.51 |
| H2-T9 : H2-T22 : H2-T17 : H2-T10 | histocompatibility 2, T region locus 9 : histocompatibility 2, T region locus 22 : histocompatibility 2, T region locus 17 : histocompatibility 2, T region locus 10 | NM_010399.2 | 2.51 |
| Mdm2 | transformed mouse 3T3 cell double minute 2 | X58876.1 | 2.50 |
| Zfx : Zfa | zinc finger protein X-linked : zinc finger protein, autosomal | NM_011768.1 | 2.49 |
| Mthfd2 | methylenetetrahydrofolate dehydrogenase (NAD+ dependent), methenyltetrahydrofolate cyclohydrolase | NM_008638.1 | 2.49 |
| Bcl6 | B-cell leukemia/lymphoma 6 | NM_009744.2 | 2.47 |
| Npn3 | neoplastic progression 3 | NM_029688.2 | 2.46 |
| Mod1 | malic enzyme, supernatant | NM_008615.1 | 2.45 |
| Ceacam1 | CEA-related cell adhesion molecule 1 | NM_011926.1 | 2.43 |
| Abcd3 | ATP-binding cassette, sub-family D (ALD), member 3 | NM_008991.1 | 2.43 |
| Tmem2 | transmembrane protein 2 | NM_031997.2 | 2.42 |
| Prkar2b | protein kinase, cAMP dependent regulatory, type II beta | NM_011158.2 | 2.42 |
| Dusp1 | dual specificity phosphatase 1 | NM_013642.1 | 2.41 |
| Mxd4 | Max dimerization protein 4 | NM_010753.2 | 2.39 |
| Impact | imprinted and ancient | NM_008378.1 | 2.39 |
| Smpd1 | sphingomyelin phosphodiesterase 1, acid lysosomal | NM_011421.1 | 2.38 |
| Aldh6a1 | aldehyde dehydrogenase family 6, subfamily A1 | NM_134042.1 | 2.37 |
| Osp94 | osmotic stress protein | NM_011020.3 | 2.37 |
| Myd116 | myeloid differentiation primary response gene 116 | NM_008654.1 | 2.37 |
| Slc2a8 | solute carrier family 2, (facilitated glucose transporter), member 8 | NM_019488.2 | 2.37 |
| Zfp367 | zinc finger protein 367 | NM_175494.2 | 2.35 |
| Nars | asparaginyl-tRNA synthetase | NM_027350.1 | 2.35 |
| Cmya1 | cardiomyopathy associated 1 | NM_011724.1 | 2.34 |
| Cat | catalase | NM_009804.1 | 2.34 |
| Gp49b | glycoprotein 49 B | NM_013532.1 | 2.34 |
| Tuft1 | tuftelin 1 | NM_011656.1 | 2.32 |
| Ceacam1 | CEA-related cell adhesion molecule 1 | NM_011926.1 | 2.32 |
| Fdps | farnesyl diphosphate synthetase | AK077979.1 | 2.32 |
| Angptl6 | angiopoietin-like 6 | NM_145154.1 | 2.30 |
| Tenc1 | tensin like C1 domain-containing phosphatase | NM_153533.1 | 2.28 |
| Serpinb12 | serine (or cysteine) proteinase inhibitor, clade B (ovalbumin), member 12 | NM_027971.1 | 2.27 |
| Mgl1 | macrophage galactose N-acetyl-galactosamine specific lectin 1 | NM_010796.1 | 2.26 |
| Mmp11 | matrix metalloproteinase 11 | NM_008606.1 | 2.26 |
| Chpt1 | choline phosphotransferase 1 | NM_144807.2 | 2.25 |
| Sparc | secreted acidic cysteine rich glycoprotein | NM_009242.1 | 2.25 |
| Gpnmb | glycoprotein (transmembrane) nmb | NM_053110.2 | 2.23 |
| Sqrdl | sulfide quinone reductase-like (yeast) | NM_021507.4 | 2.23 |
| Pfkm | phosphofructokinase, muscle | NM_021514.2 | 2.22 |
| Pdcd4 | programmed cell death 4 | NM_011050.1 | 2.22 |
| Picalm | phosphatidylinositol binding clathrin assembly protein | NM_146194.2 | 2.22 |
| Bat4 | HLA-B associated transcript 4 | NM_032460.1 | 2.22 |
| Aplp2 | amyloid beta (A4) precursor-like protein 2 | M97216.1 | 2.21 |
| Zfp101 | zinc finger protein 101 | NM_009542.1 | 2.21 |
| Traf4 | Tnf receptor associated factor 4 | NM_009423.2 | 2.20 |
| Zfp336 | zinc finger protein 336 | NM_028986.1 | 2.20 |
| Cyp2c55 | cytochrome P450, family 2, subfamily c, polypeptide 55 | NM_028089.1 | 2.20 |
| Col4a3bp | procollagen, type IV, alpha 3 (Goodpasture antigen) binding protein | NM_023420.1 | 2.20 |
| Hebp1 | heme binding protein 1 | AF117613.1 | 2.19 |
| Slc25a30 | solute carrier family 25, member 30 | AK090086.1 | 2.19 |
| Ass1 | argininosuccinate synthetase 1 | NM_007494.2 | 2.17 |
| 4921511C16 | hypothetical protein 4921511C16 | NM_183307.1 | 2.17 |
| Tec | cytoplasmic tyrosine kinase, Dscr28C related (Drosophila) | NM_013689.2 | 2.17 |
| Rnf13 | ring finger protein 13 | NM_011883.1 | 2.17 |
| Zfp68 | zinc finger protein 68 | NM_013844.1 | 2.16 |
| Alas1 | aminolevulinic acid synthase 1 | NM_020559.1 | 2.16 |
| Gcl | germ cell-less homolog (Drosophila) | NM_011818.2 | 2.14 |
| Twsg1 | twisted gastrulation homolog 1 (Drosophila) | NM_023053.1 | 2.14 |
| Nical | NEDD9 interacting protein with calponin homology and LIM domains | NM_138315.1 | 2.14 |
| Sep-06 | septin 6 | NM_019942.2 | 2.14 |
| Scp2 | sterol carrier protein 2, liver | NM_011327.1 | 2.14 |
| Tmc4 | transmembrane channel-like gene family 4 | NM_181820.1 | 2.14 |
| Zfp108 | zinc finger protein 108 | NM_018791.1 | 2.13 |
| Tnfaip2 | tumor necrosis factor, alpha-induced protein 2 | NM_009396.1 | 2.13 |
| Rbbp9 | retinoblastoma binding protein 9 | NM_015754.1 | 2.13 |
| Sdcbp | syndecan binding protein | NM_016807.1 | 2.13 |
| Abcb1b | ATP-binding cassette, sub-family B (MDR/TAP), member 1B | NM_011075.1 | 2.13 |
| Akr1b3 | aldo-keto reductase family 1, member B3 (aldose reductase) | NM_009658.2 | 2.13 |
| Slc11a1 | solute carrier family 11 (proton-coupled divalent metal ion transporters), member 1 | NM_013612.1 | 2.13 |
| Olfr1395 | olfactory receptor 1395 | NM_146877.1 | 2.12 |
| Lrrc1 | leucine rich repeat containing 1 | NM_172528.2 | 2.11 |
| Pigh | phosphatidylinositol glycan, class H | NM_029988.1 | 2.11 |
| Acat3 | acetyl-Coenzyme A acetyltransferase 3 | NM_153151.1 | 2.09 |
| Cbx1 | chromobox homolog 1 (Drosophila HP1 beta) |  | 2.09 |
| Slc30a9 | solute carrier family 30 (zinc transporter), member 9 | NM_178651.2 | 2.09 |
| Hspd1 | heat shock protein 1 (chaperonin) | NM_010477.2 | 2.09 |
| Gpsm1 | G-protein signalling modulator 1 (AGS3-like, C. elegans) | NM_153410.2 | 2.08 |
| Pqlc2 | PQ loop repeat containing 2 | NM_145384.1 | 2.08 |
| Pycr1 | pyrroline-5-carboxylate reductase 1 | NM_144795.1 | 2.08 |
| Esd | esterase D/formylglutathione hydrolase | NM_016903.2 | 2.08 |
| Uxs1 | UDP-glucuronate decarboxylase 1 | NM_026430.1 | 2.07 |
| Slc27a4 | solute carrier family 27 (fatty acid transporter), member 4 | NM_011989.1 | 2.07 |
| Fcgrt | Fc receptor, IgG, alpha chain transporter | NM_010189.1 | 2.06 |
| Abcd4 | ATP-binding cassette, sub-family D (ALD), member 4 | NM_008992.1 | 2.05 |
| Tpmt | thiopurine methyltransferase | NM_016785.1 | 2.05 |
| Rad23b | RAD23b homolog (S. cerevisiae) | NM_009011.2 | 2.04 |
| Zfp146 | zinc finger protein 146 | NM_011980.1 | 2.04 |
| Fcgr2b | Fc receptor, IgG, low affinity IIb | NM_010187.1 | 2.04 |
| Clk | CDC-like kinase | NM_009905.1 | 2.04 |
| Nfe2l1 | nuclear factor, erythroid derived 2,-like 1 | NM_008686.2 | 2.03 |
| Nipsnap3b | nipsnap homolog 3B (C. elegans) | NM_025623.1 | 2.03 |
| Mpv17l | Mpv17 transgene, kidney disease mutant-like | NM_033564.1 | 2.02 |
| Btg1 | B-cell translocation gene 1, anti-proliferative | NM_007569.1 | 2.02 |
| Setmar | SET domain and mariner transposase fusion gene | NM_178391.2 | 2.02 |
| Sla | src-like adaptor | NM_009192.1 | 2.01 |
| Abhd4 | abhydrolase domain containing 4 | NM_134076.1 | 2.01 |
| Sdh1 | sorbitol dehydrogenase 1 | NM_146126.1 | 2.01 |
| Tnfrsf11a | tumor necrosis factor receptor superfamily, member 11a | NM_009399.2 | -2.01 |
| Zcchc11 | zinc finger, CCHC domain containing 11 | NM_175472.2 | -2.02 |
| Ssbp1 | single-stranded DNA binding protein 1 | NM_212468.3 | -2.02 |
| Tal1 | T-cell acute lymphocytic leukemia 1 | NM_011527.1 | -2.02 |
| Ctdspl | CTD (carboxy-terminal domain, RNA polymerase II, polypeptide A) small phosphatase-like | NM_133710.1 | -2.03 |
| Pabpc1 | poly A binding protein, cytoplasmic 1 | NM_008774.2 | -2.07 |
| Mbtps1 | membrane-bound transcription factor protease, site 1 | AK002809.1 | -2.07 |
| Arid3a | AT rich interactive domain 3A (Bright like) | NM_007880.1 | -2.13 |
| Lfng | lunatic fringe gene homolog (Drosophila) | AK004642.1 | -2.13 |
| Prtn3 | proteinase 3 | NM_011178.2 | -2.14 |
| Plk1 | polo-like kinase 1 (Drosophila) | NM_011121.2 | -2.15 |
| U2af1-rs2 | U2 small nuclear ribonucleoprotein auxiliary factor (U2AF) 1, related sequence 2 | NM_178794.2 | -2.17 |
| Pes1 | pescadillo homolog 1, containing BRCT domain (zebrafish) | NM_022889.2 | -2.19 |
| Fau | Finkel-Biskis-Reilly murine sarcoma virus (FBR-MuSV) ubiquitously expressed (fox derived) | NM_007990.1 | -2.24 |
| Cks2 | CDC28 protein kinase regulatory subunit 2 | NM_025415.1 | -2.25 |
| Wasf2 | WAS protein family, member 2 | NM_153423.3 | -2.26 |
| Nvl | nuclear VCP-like | NM_026171.1 | -2.27 |
| Mrpl34 | mitochondrial ribosomal protein L34 | NM_053162.1 | -2.31 |
| Fkbp11 | FK506 binding protein 11 | NM_024169.2 | -2.31 |
| Gpr35 | G protein-coupled receptor 35 | NM_022320.2 | -2.34 |
| Ank2 | ankyrin 2, brain | NM_178655.2 | -2.35 |
| Scgf | stem cell growth factor | NM_009131.1 | -2.37 |
| Timm13a | translocase of inner mitochondrial membrane 13 homolog a (yeast) | NM_013899.1 | -2.41 |
| Ptgs1 | prostaglandin-endoperoxide synthase 1 | NM_008969.1 | -2.41 |
| Cks2 | CDC28 protein kinase regulatory subunit 2 | NM_025415.1 | -2.41 |
| Tle6 | transducin-like enhancer of split 6, homolog of Drosophila E(spl) | NM_053254.1 | -2.43 |
| Mtf1 | metal response element binding transcription factor 1 | NM_008636.2 | -2.44 |
| Olr1 | oxidized low density lipoprotein (lectin-like) receptor 1 | NM_138648.1 | -2.47 |
| Pilra | paired immunoglobin-like type 2 receptor alpha | NM_153510.1 | -2.51 |
| Ctsw | cathepsin W | NM_009985.2 | -2.58 |
| Timm9 | translocase of inner mitochondrial membrane 9 homolog (yeast) | NM_013895.2 | -2.59 |
| Gpr84 | G protein-coupled receptor 84 | NM_030720.1 | -2.67 |
| Tbxa2r | thromboxane A2 receptor | NM_009325.1 | -2.69 |
| Cxcl14 | chemokine (C-X-C motif) ligand 14 | AK004615.1 | -2.73 |
| Snrpg | small nuclear ribonucleoprotein polypeptide G | NM_026506.1 | -2.73 |
| Rps27a | ribosomal protein S27a | NM_024277.1 | -2.83 |
| Ltb | lymphotoxin B | NM_008518.1 | -2.86 |
| Hoxa7 | homeo box A7 | NM_010455.1 | -2.88 |
| Uqcr | ubiquinol-cytochrome c reductase (6.4kD) subunit | NM_025650.1 | -3.00 |
| Hip1 | huntingtin interacting protein 1 | NM_146001.1 | -3.38 |

The cross-talk between hypoxia and NO generated 162 transcripts unique to treatment of cells with the combination of hypoxia and NO. Hyp = hypoxic treatment (1% O_2_); NO = nitric oxide treatment (0.5 mM DETA-NO).
